# Multidimensional Tuning in Motor Cortical Neurons during Active Behavior

**DOI:** 10.1523/ENEURO.0109-20.2020

**Published:** 2020-07-29

**Authors:** Rachel C. Yuan, Sarah W. Bottjer

**Affiliations:** 1 Neuroscience Graduate Program, University of Southern California, Los Angeles, CA 90089; 2 Section of Neurobiology, University of Southern California, Los Angeles, CA 90089

**Keywords:** motor cortex, multimodal, sensorimotor, songbird

## Abstract

A region within songbird cortex, dorsal intermediate arcopallium (AId), is functionally analogous to motor cortex in mammals and has been implicated in song learning during development. Non-vocal factors such as visual and social cues are known to mediate song learning and performance, yet previous chronic-recording studies of regions important for song behavior have focused exclusively on neural activity in relation to song production. Thus, we have little understanding of the range of non-vocal information that single neurons may encode. We made chronic recordings in AId of freely behaving juvenile zebra finches and evaluated neural activity during diverse motor behaviors throughout entire recording sessions, including song production as well as hopping, pecking, preening, fluff-ups, beak interactions, scratching, and stretching. These movements are part of natural behavioral repertoires and are important components of both song learning and courtship behavior. A large population of AId neurons showed significant modulation of activity during singing. In addition, single neurons demonstrated heterogeneous response patterns during multiple movements (including excitation during one movement type and suppression during another), and some neurons showed differential activity depending on the context in which movements occurred. Moreover, we found evidence of neurons that did not respond during discrete movements but were nonetheless modulated during active behavioral states compared with quiescence. Our results suggest that AId neurons process both vocal and non-vocal information, highlighting the importance of considering the variety of multimodal factors that can contribute to vocal motor learning during development.

## Significance Statement

Motor cortex across taxa receives highly integrated, multimodal information and has been implicated in both execution and acquisition of complex motor skills, yet studies of motor cortex typically employ restricted behavioral paradigms that target select movement parameters, preventing wider assessment of the diverse sensorimotor factors that can affect motor cortical activity. Recording in dorsal intermediate arcopallium (AId) of freely behaving juvenile songbirds that are actively engaged in sensorimotor learning offers unique advantages for elucidating the functional role of motor cortical neurons. The results demonstrate that a diverse array of factors modulate motor cortical activity and lay important groundwork for future investigations of how multimodal information is integrated in motor cortical regions to contribute to learning and execution of complex motor skills.

## Introduction

Goal-directed skill learning underlies our ability to acquire motor skills and flexibly perform them in response to changing environmental contexts. Both learning and performance of motor skills are highly sensorimotor processes: successful acquisition of motor behaviors entails integration across internal and external sources of sensory feedback to guide accurate refinement of motor output. Correspondingly, motor cortical neurons demonstrate multidimensional tuning that reflects integration across a variety of inputs: motor cortical neurons involved in performance of skilled behaviors have been shown to encode not only motor parameters (e.g., movement force or direction) but also non-motor parameters such as preparatory activity before movement execution or visual information specific to a target object’s location in object-directed reaching tasks (Tanji and Evarts, 1976; Evarts and Fromm, 1977; Murata et al., 1997; Shen and Alexander, 1997; Ferezou et al., 2007). In addition, increasing evidence indicates that motor cortex serves not only as a driver of learned movements but also as a central locus for the acquisition of new motor skills across a variety of movements and training paradigms (Whishaw et al., 1991; Whishaw, 2000; Darling et al., 2011; Guo et al., 2015; Kawai et al., 2015; Makino et al., 2016; Peters et al., 2017; Papale and Hooks, 2018; Hwang et al., 2019). These findings suggest motor cortex as a dynamic substrate that actively integrates diverse streams of information to contribute to sensorimotor learning and performance. However, the potential influence of various multimodal inputs on motor cortical activity during behavior is difficult to assess in anesthetized and/or restrained experimental paradigms that focus on a single motor task; recordings in freely behaving animals, especially in the context of sensorimotor skill learning, afford opportunities for investigating sensorimotor integration in motor cortical neurons (Ebbesen et al., 2017; Zhang et al., 2017; Mimica et al., 2018).

Vocal learning in juvenile songbirds entails integration of social cues as well as visual, auditory, and somatosensory information to guide refinement of variable babbling into stereotyped song (Price, 1979; West and King, 1988; Eales, 1989; Mann et al., 1991; Mann and Slater, 1995; King et al., 2005; Derégnaucourt et al., 2013; Chen et al., 2016; Ljubičić et al., 2016; Carouso-Peck and Goldstein, 2019). Moreover, song production occurs in the context of other social behaviors such as hopping, beak interactions, and preening. For example, adult males must combine both vocal and non-vocal elements in an integrated performance to successfully court females (Morris, 1954; Williams, 2001; Cooper and Goller, 2004; Tomaszycki and Adkins-Regan, 2005; Dalziell et al., 2013; Ota et al., 2015; Ullrich et al., 2016). Thus, songbirds offer a model system for examining multiple diverse behaviors during acquisition and performance of a complex motor skill. However, despite evidence for the importance of non-vocal factors in song learning and performance, few studies of regions that process song-related information have examined neural activity in relation to non-vocal behaviors.

**Figure 1.**
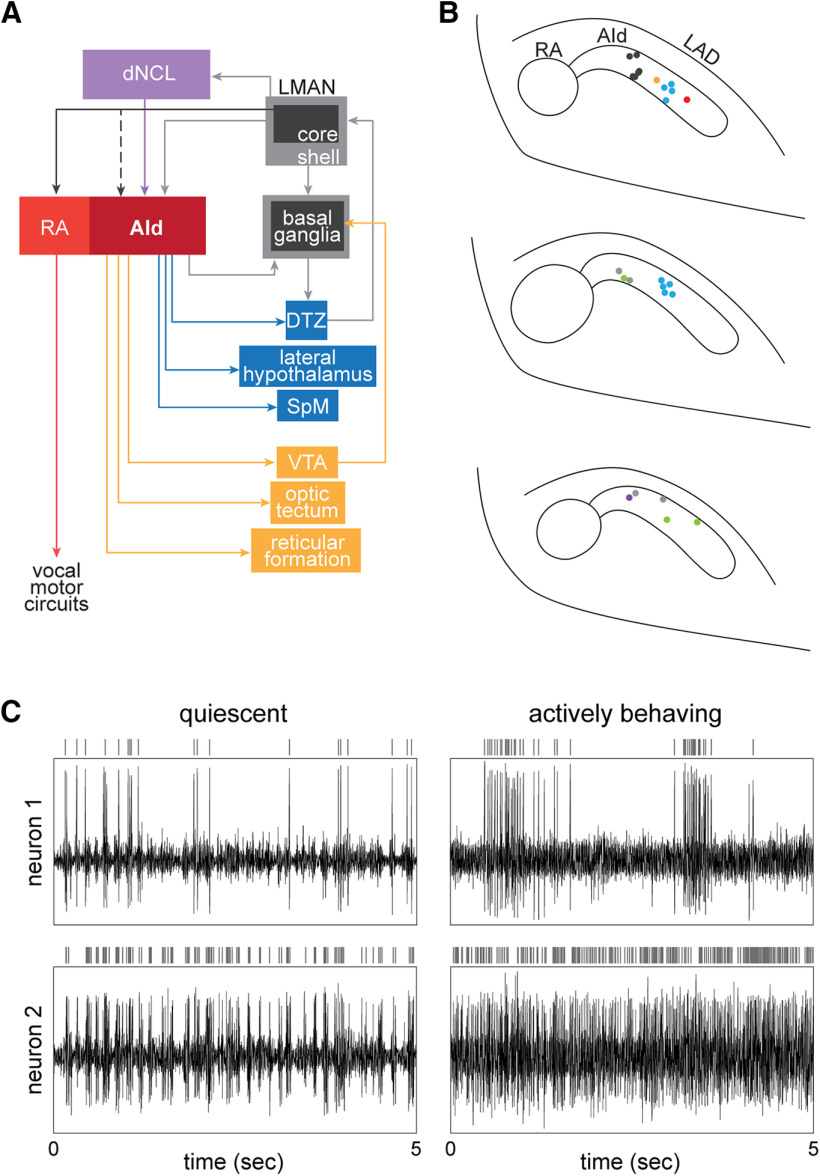
AId neurons are well situated to integrate multimodal inputs and distribute information across various cortical-subcortical circuits. ***A***, AId receives inputs from upstream cortical regions LMAN-shell and dNCL. LMAN-shell is part of a cortico-basal ganglia loop that mediates vocal learning, whereas dNCL receives inputs from LMAN-shell as well as multiple pathways processing somatosensory, visual, and auditory information. AId of juvenile birds also receives inputs from LMAN-core via axon collaterals of LMAN-core→RA neurons that drive vocal output; robust collaterals are present in juvenile birds before ∼40–45 dph but are not present in older juvenile or adult birds. Projections from AId to striatum and several midbrain and thalamic regions give rise to both feedback and feed-forward pathways, creating several opportunities for information transfer between cortical and subcortical regions. DTZ, dorsal thalamic zone (includes both DLM and DMP); LMAN, lateral magnocellular nucleus of the anterior nidopallium; RA, robust nucleus of the arcopallium. ***B***, top to bottom, Caudal-to-rostral series of coronal sections 250 μm apart mapping locations of recordings made in AId. Different colored circles represent sites recorded from different birds (*n* = 7). ***C***, Raw traces of extracellular activity simultaneously recorded at two different sites within AId of a juvenile bird (44 dph) while the bird was resting (left column; “quiescent”) versus hopping around the recording cage (right column; “actively behaving”). Vertical lines above each raw activity trace indicate spikes from a single neuron sorted from the extracellular activity.

In songbirds, the dorsal intermediate arcopallium (AId) has been considered to be analogous to motor cortex in mammals and has been implicated in both vocal learning and non-vocal motor behavior (Feenders et al., 2008; Bottjer and Altenau, 2010; Karten, 2013). AId receives inputs that process multimodal sensory information via dorsal caudolateral nidopallium (dNCL) as well as information from cortico-basal ganglia circuitry that mediates vocal learning (via LMAN-shell) and, in turn, makes a variety of projections that give rise to feedforward and feedback pathways through subcortical and brainstem regions (Fig. 1*A*; Zeier and Karten, 1971; Bottjer et al., 2000; Paterson and Bottjer, 2017). AId is thus well suited to integrate multiple sources of external and internal sensory information to contribute to motor skill learning and performance.

We made chronic recordings in AId of freely behaving juvenile songbirds during the sensorimotor period of vocal learning and analyzed the activity of single neurons during singing as well as during several discrete behaviors performed as part of the natural repertoire of zebra finches. This novel approach enabled us to explore the idea that neural activity patterns in brain regions that mediate vocal learning are not restricted to song production. Our results represent an extensive assessment of motor cortical activity across a wide variety of natural behaviors, thereby informing our understanding of how these neurons may contribute to motor skill learning and production.

## Materials and Methods

### Subjects

All animal procedures were performed in accordance with the University of Southern California animal care committee’s regulations. Seven male juvenile zebra finches [43–58 days post-hatch (dph); mean age 46 dph on first day of recording] were used; this age range corresponds to the early stages of the sensorimotor learning period, when birds have completed memorization of vocal sounds from social tutors and are producing immature, variable vocalizations. Female zebra finches do not produce song and were therefore excluded from this study. All birds were raised in group aviaries until at least 33 dph, remaining with their natural parents and thereby receiving normal auditory and social experience during the tutor memorization period (Immelmann, 1969; Böhner, 1983, 1990; Eales, 1985; Mann and Slater, 1995; Roper and Zann, 2006). Juveniles were separated from group aviaries at 33–35 dph and housed in single cages within the experimental rig. Each bird’s tutor was placed in a separate cage within view of the juvenile to help it acclimate to the experimental rig for 2–5 d before the start of recording.

### Anatomy

We refer to our region of interest as AId, following the terminology of Reiner et al. (2004). AId extends laterally from the vocal motor nucleus RA (robust nucleus of the arcopallium) and is coterminous with RA. AId receives direct input from both LMAN-shell and dNCL and projects to several downstream targets, including the ventral tegmental area (VTA; Fig. 1*A*; Bottjer et al., 2000). Many sources of afferent input converge in the intermediate arcopallium and different arcopallial subregions in turn make a variety of downstream projections, resulting in a complex and heterogeneous area. It seems possible that AId as defined by afferent inputs from LMAN-shell may overlap slightly with AIV, which was defined by Mandelblat-Cerf et al. (2014) as an area of intermediate arcopallium that receives inputs from higher-level auditory cortical regions and projects to VTA (Gale et al., 2008). In addition, AIV clearly overlaps with or is identical to a region known as “RA cup,” which is a putative auditory area that is located primarily ventral and anterior to RA (Kelley and Nottebohm, 1979; Vates et al., 1996; Mello et al., 1998; Yuan and Bottjer, 2019). We discuss the high degree of anatomic complexity in the arcopallial regions surrounding RA and AId in a note to the paper by Mandelblat-Cerf et al. (2014). While such anatomic complexities remain to be clarified, we provide schematics of our recording locations in Figure 1*B* to illustrate the region of arcopallium we refer to as AId.

Throughout the text, we refer to AId as analogous in function and connectivity to motor cortex, using the term “cortex” in a generic sense as described by Reiner et al. (2004; p. 395) as including the part of telencephalon that is “pallial in nature and therefore homologous as a field to the brain region of mammals that includes the neocortex, claustrum, and pallial amygdala.”

### Electrophysiology

At 35–40 dph, birds were anesthetized with 1.5% isoflurane (inhalation) and placed in a stereotaxic instrument. An electrode assembly consisting of four tetrodes affixed to a movable microdrive was fixed to the skull using C&B Metabond (Parkell), such that the tetrodes were implanted 500 μm dorsal to AId. Each tetrode consisted of four twisted polyimide-coated Nichrome wires (0.012-mm diameter Redi Ohm 800, Kanthal) routed through fused silica capillary tubing and electroplated with non-cyanide gold plating solution (SIFCO 5355). One day after surgery, the tetrode assembly was connected to a recording headstage (HS-16, Neuralynx) with a flexible cable connected to a commutator (PSR, Neuralynx); 15 channels of neural data were amplified, band passed between 300 and 5000 Hz (Lynx-8, Neuralynx), and digitized at 32 kHz using Spike2 software (Power 1401 data acquisition interface, CED). Audio and video were recorded coincident with neural activity: vocalizations were recorded with a lavalier microphone (Sanken COS-11D) mounted in the cage, and a USB-video camera (30 FPS, ELP) was placed at the front of the cage to record video. Consecutive 30-min recordings were made from 7 A.M. to 6 P.M. each day. Tetrodes were manually advanced with the microdrive when the cells being recorded were lost or had already been recorded for at least 2 d, as indicated by stability and consistency of the extracellular signal. At the end of each experiment, birds were perfused (0.7% saline followed by 10% formalin), and brains were removed and postfixed for 72 h before being cryo-protected (30% sucrose solution) and frozen-sectioned (50 μm thick). Sections were Nissl stained with thionin to visualize tetrode tracks and verify recording locations. Sites within 50 μm from the border of AId were considered to be within AId if neural activity matched characteristic AId firing (intermittent periods of high firing or a high rate of tonic activity during active behavior; Fig. 1*C*).

Movement artifact in neural recordings was correlated across recording channels and was eliminated or reduced using offline common average referencing: for each recording channel, the signal across ∼8–14 remaining recording channels was averaged and subtracted from that channel to remove movement artifact (Ludwig et al., 2009). Channels were visually inspected after referencing to ensure that spiking activity was not distorted. After common average reference subtraction, single units were sorted from multiunit data by first automatically clustering units with KlustaKwik (KD Harris, University College London). KlustaKwik clusters were manually inspected across 18 different waveform features and further refined using MClust (A. D. Redish, University of Minnesota). Clusters were considered for analysis only if the signal-to-noise ratio was >2 and <1% of spikes had an interspike interval (ISI) < 2 ms.

### Behavioral scoring

Sixteen 30-min sessions were recorded across seven birds (median of three sessions per bird). Videos from recording sessions were scored for movements and state periods using ELAN (The Language Archive, Max Planck Institute for Psycholinguistics; Movies 1, 2, 3). We scored each single occurrence of pecks, hops, preening behavior, beak interactions (beak wipes and periods when the bird’s beak was in contact with perches, food cup edges, etc. for longer than the duration of a peck), fluff-ups, scratches, and stretches. Head and postural movements occurred so frequently that it was impractical to score all of them for all cells. These movements did not occur concurrently with any of the seven scored movements, but did occur during singing. To test whether head movements contributed to singing activity, we scored head movements that occurred during singing and during 30 s of non-singing before and after each singing episode in a subset of 36 singing-responsive neurons. In addition, all head and postural movements were scored for 12 neurons across two recording sessions in two birds.

**Movie 1.** Example video of a juvenile zebra finch demonstrating the seven scored movements: pecks, beak interactions, preening, hopping, stretching, scratching, and fluff-ups. See Movie 2 for examples of pecking movements during eating versus non-eating periods.

**Movie 2.** Example video of a juvenile zebra finch during periods of active behavior, singing, and eating.

**Movie 3.** Example video of neural activity recorded on a single channel while the juvenile zebra finch hopped around the cage, demonstrating firing rate increases whenever the bird hopped towards the left side of the cage. This example suggests the possibility that hopping-related activity was context- or location-dependent, but we did not have enough examples to test this idea. Vertical lines above the raw activity trace indicate spikes from a single neuron sorted from the extracellular activity.

In addition to scoring discrete movements, we developed a novel way of measuring behavior throughout recording sessions: each session was segmented into contiguous time periods that were classified into one of five behavioral “state” periods based on the bird’s behavior: eating, singing, active-movement, quiet-attentive, or quiescent; these state periods tiled the entire duration of the recording session (Fig. 6*A*). Eating states were defined as periods during which the bird was pecking at seed or grit, hulling or ingesting seeds, or pausing in between these behaviors for at most 1 s. Although pecks occurred most often during eating, eating states could also include other scored movements such as hops or preening, or unscored movements such as head movements as long as they occurred within the brief (≤1 s) pauses that occurred while birds were actively eating. Singing states were defined by song behavior; they began whenever the bird produced song and lasted as long as song syllables continued to occur within 1 s of each other (intersyllable interval ≤ 1 s). Birds often made head movements during singing and occasionally made scored movements such as hopping or pecking in between bouts of singing. Active-movement states were defined as non-eating and non-singing periods during which the bird made active movements with pauses of at most 1 s in between movements; these periods could include any of the seven movements that were scored as well as head and postural body movements that were not scored. Quiet-attentive states were defined by times when the bird was not eating, singing, or moving around the cage for >1 s; they continued as long as the bird made at most small head movements and otherwise remained unmoving but alert. Quiescent periods were defined as periods during which the bird was completely still and not obviously paying attention to any stimulus, with eyes partially or fully closed. Quiescent state times were segmented into 1-s intervals that were used as baseline periods for analyses of scored movements (see “Data analysis” section below).

### Data analysis

To test for significant responses during scored movements, firing rates across occurrences of each movement type were compared against quiescence. Quiescent baseline periods were generated by dividing quiescent state periods (as described in “Behavioral scoring” section above) into 1-s segments. The firing rate during two 1-s quiescent segments that occurred closest in time to each movement occurrence was used as a corresponding baseline. Fourteen neurons were recorded during sessions that lacked quiescent state periods. For these 14 neurons, 1-s baseline periods were taken from times within quiet-attentive state periods when the bird was verified to be unmoving (although clearly alert, unlike in quiescent state). To compare movement responses across neurons, standardized response strength (RS) was calculated for each movement type as:

$$standardized\;response\;str\text{e}ngth=\frac{{\bar{FR}}_{m}-{\bar{FR}}_{b}}{\sqrt{Var\left(FR_{m}\right)+Var\left(FR_{b}\right)-2\times Covar(FR_{m},FR_{b})}},$$

where FR_m_ is the firing rate during movement occurrences and FR_b_ is the firing rate during corresponding baseline periods. A positive value indicates an increase in firing rate during the movement compared with quiescence whereas a negative value indicates a decrease in firing rate during movement. This measure is referred to as RS throughout the text. Mean RS values across neurons are reported as the mean ± SEM.

To test for changes in activity around movement onsets, for each neuron we generated a 25-ms bin histogram of the spiking response across all occurrences of the movement; histogram windows were 1 s long and centered on movement onsets. Spike times during each movement repetition were shuffled to obtain a resulting histogram of shuffled spike data; this was repeated 1000 times, resulting in 1000 histograms of shuffled data. Each bin of the actual spike data histogram was considered significantly excited if the count in that bin was >95% of maximum values from the shuffled dataset; likewise, the bin was considered significantly suppressed if the count was lower than 95% of minimum values from the shuffled dataset. Onset responses were defined as responses that contained at least two consecutive bins (50 ms) of significant maxima or minima within 100 ms of movement onset.

We tested for significant offset-aligned responses using the same method and parameters as onset-aligned responses, except that the 1-s windows used for histograms of actual and shuffled data were centered around movement offsets; offset responses were defined as responses that contained at least two consecutive bins (50 ms) of significant maxima or minima within 100 ms of movement offset. Because of the short duration of pecks and hops (mean hop duration = 0.28 s; mean peck duration = 0.25 s), it was possible for the same maxima or minima to be captured in both onset-aligned and offset-aligned responses. To ensure that offset-aligned activity could be accurately distinguished, for these movements, we only counted excited or suppressed offset responses that did not demonstrate significant changes in onset-aligned activity. Preening movements were relatively long in duration (mean duration = 1.5 s), so all preening-aligned offset responses were counted.

We tested for significant modulation of firing rate during states by dividing all state periods in each recording session into 1-s segments and calculating the average firing rate during each segment for each neuron. We compared the distributions of average firing rates across segments for each state against quiescence to determine whether activity was significantly increased or decreased during non-quiescent states for each neuron. Fourteen neurons were recorded during sessions that lacked quiescent state periods and were excluded from these analyses.

As an additional means of characterizing firing rate modulation during states, we defined “events,” brief periods of excitation and suppression, from histograms of spiking activity. We segmented the instantaneous firing rate (IFR) across each recording session into 10-ms bins and smoothed the IFR with a moving average filter (span = 3 bins). Excited events were defined as periods during which the smoothed IFR across five or more 10-ms bins (50 ms or more) exceeded the average firing rate across quiescent state periods by ≥3 SDs. Suppressed events were defined as periods during which the smoothed IFR across five or more 50-ms bins fell below the average firing rate across quiescent state periods by ≤1.5 SDs. To compare across neurons, the number of events in each state was normalized by dividing the number of excited or suppressed events in each state by the total duration of that state for each neuron.

### Statistics

Movement responses were tested for significance against quiescent baselines (see “Data analysis” section above) using Wilcoxon signed-rank tests; Benjamini–Hochberg *post hoc* tests were used to apply corrections for multiple comparisons (Benjamini and Hochberg, 1995). Neurons that demonstrated a significant difference between movement and baseline for at least one scored movement were considered movement-responsive. To test whether movement responses were context-selective, RSs during movements from one context versus another context were compared using Mann–Whitney tests for each neuron (for example, comparing pecks during eating vs non-eating periods, and comparing head movements during singing vs non-singing periods). Mann–Whitney tests were also used to compare RSs during singing periods with versus without head movements in individual neurons. We were not able to use signed-rank tests in these cases because of different numbers of observations in the comparisons. χ^2^ tests were run to compare proportions between more than two groups (for example, proportions of neurons that were responsive during each movement type). In case of significance, Fisher’s exact tests were used as a follow-up to make pairwise comparisons of proportions between groups, and Benjamini–Hochberg *post hoc* tests were used to apply a correction for multiple comparisons. Binomial tests were used to judge whether the relative proportions of excited versus suppressed responses among movement-responsive neurons were different from chance. Comparisons of firing rate distributions between each state and quiescence (see “Data analysis” section above) were made using Kolmogorov–Smirnov tests, with a Benjamini–Hochberg *post hoc* test applied for multiple comparisons. Measures between different state periods (ISIs, normalized number of events, normalized firing rates among unresponsive neurons) were compared using sets of pairwise linear contrasts based on trimmed means (20% trimming); this linear contrast method has been shown to be robust to common assumption violations such as non-normality and heteroscedasticity (Wilcox and Serang, 2017). For all tests, *p* < 0.05 was considered significant.

## Results

We made extracellular recordings from 119 neurons in AId of freely behaving juvenile zebra finches (43–58 dph) housed singly in a recording cage as they actively engaged in sensorimotor vocal practice. Figure 1*B* illustrates locations of recording sites in AId. A typical 30-min recording period included various overt behaviors and periods of quiescence when the bird was not moving. To investigate how neural activity in AId corresponds to different behaviors, we scored seven different movements during each recording that could be identified reliably: pecks, hops, preening episodes, beak interactions with objects in the recording cage (e.g., beak wipes or non-peck interactions with cage bars), fluff-ups, stretches, and scratching episodes; we also marked periods of singing (Movies 1, 2, 3). We developed a novel approach in which we examined spiking patterns of single neurons throughout each recording period to investigate whether AId neurons are selective for different movement types and/or singing behavior in juvenile birds.

### Responsivity of AId neurons during movements

Patterns of spiking were highly variable across individual neurons, ranging from phasic to tonic activity. In addition, each neuron’s activity was highly modulated throughout a typical recording session, showing either excitation and/or suppression during different movements. Figure 1*C* illustrates two different neurons recorded in one bird while it was quiescent (no overt movements, left columns) and while it hopped around the cage (right columns). The neuron in the top panel fired intermittently in small bursts of at most three spikes during quiescence, while the neuron in the bottom panel exhibited dense bursting activity. As the bird hopped around the cage, the neuron in the top panel shifted to longer periods of high firing separated by relative inactivity while the neuron in the bottom panel shifted to a high tonic rate of firing. To investigate whether such modulations were related to specific movements, for each neuron we compared firing rates during different movement types against baseline firing rates during quiescent periods that were closest in time to each movement occurrence (see Materials and Methods). To compare movement-related activity across neurons, we calculated the response strength of each neuron during each movement type, defined as the standardized difference in average firing rate during each movement type versus baseline periods (see Materials and Methods).

**Figure 2.**
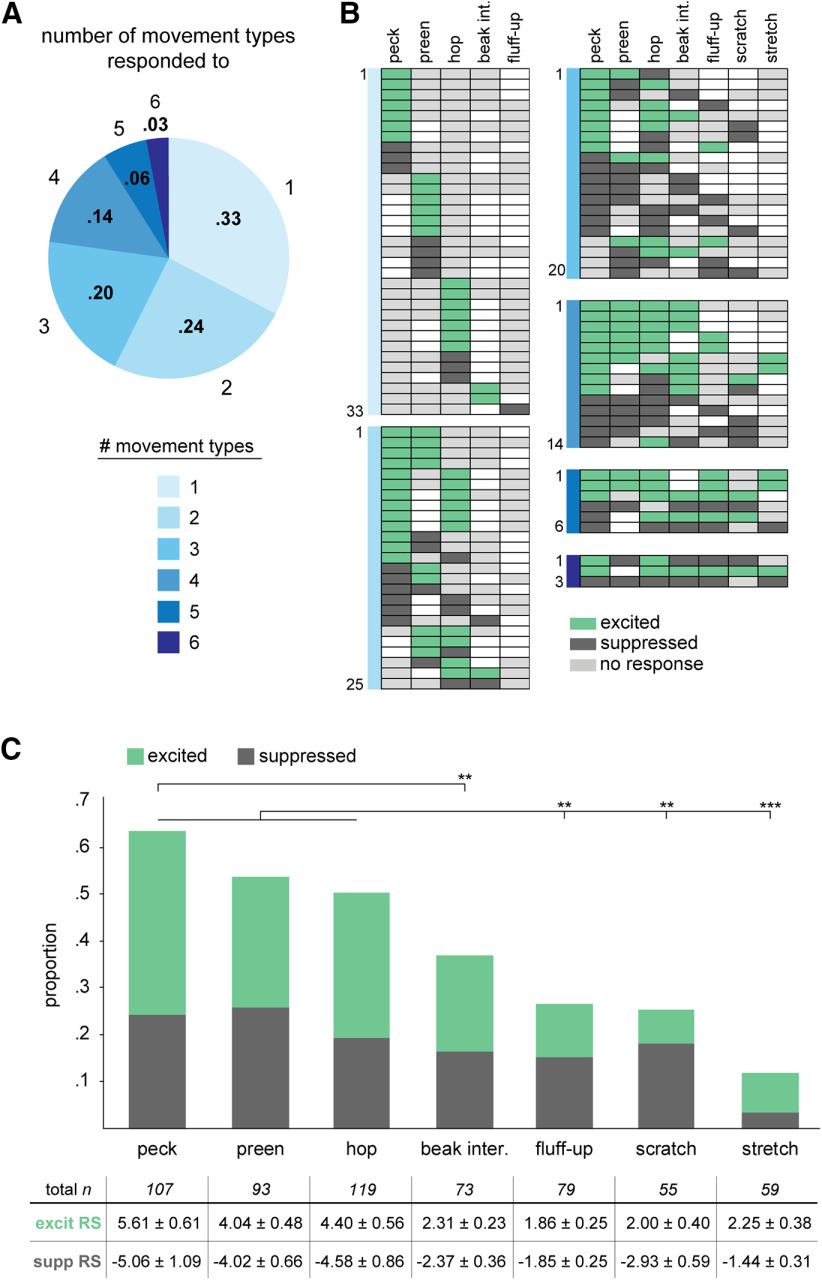
AId neurons respond during different scored movements with excitation and/or suppression. ***A***, Proportions of single AId neurons that responded during different numbers of movements. 33/101 movement-responsive neurons responded during one movement, 25/101 during two, 20/101 during three, 14/101 during four, 6/101 during five, and 3/101 during six movements. ***B***, Each row of each chart indicates movements during which each neuron was excited (green), suppressed (dark gray), or not responsive (light gray). Unfilled boxes indicate that no data during that movement was recorded for that neuron. Charts are grouped according to colors in ***A***, based on the number of movements during which neurons responded. ***C***, Proportions of AId neurons that were significantly excited (green) or suppressed (dark gray) during each movement type. Table below indicates the number of neurons recorded during each movement type and the corresponding excited and suppressed RSs (mean standardized RS ± SEM); **p <* *0.005, ***p <* *0.0001.

The majority of AId neurons exhibited a significant change in firing rate during at least one movement type compared with quiescent baseline periods and were thus classified as “movement responsive” (101 out of 119 neurons, 85%). Among 101 movement-responsive neurons, 33 (33%) responded during only one scored movement, whereas 68 (67%) responded during two or more movements (Fig. 2*A*). Few cells responded during five or six movements, and no cells responded during all seven movements. Figure 2*B* depicts the range of responsivity in these 101 neurons. For example, 10 neurons were either excited or suppressed during pecks but were otherwise not responsive during any of the other six scored movements; likewise, two other groups of 10 neurons each selectively modulated their firing rate only during preening or hops. Neurons modulated during multiple movements showed heterogeneous responsivity: single neurons could demonstrate excitation during some movement types and suppression during others, and responsivity profiles across neurons included different subsets of movement types.

Higher proportions of neurons showed altered firing rates during pecks, preening, and/or hops compared with other movements: 68 out of 107 neurons (64%) were significantly modulated during pecks, 50 out of 93 (54%) during preening, and 60 out of 119 (50%) during hops (Fig. 2*C*). These proportions did not differ (Fisher’s exact test, Benjamini–Hochberg corrected, peck vs preen *p* = 0.23, peck vs hop *p* = 0.09, preen vs hop *p* = 0.71) and were each greater than the proportions of neurons that responded during fluff-ups (21/79, 27%), scratches (14/55, 25%), and stretches (7/59, 12%; Fisher’s exact test, Benjamini–Hochberg corrected, *p* < 0.05 for comparisons between pecks, hops, and preening against each of the other three movements; *p* > 0.05 for comparisons among these latter three movements; Fig. 2*C*). In addition to the seven scored movements, birds constantly made quick, saccade-like movements throughout recording periods, resulting in over a thousand head or postural movements in a typical 30-min session. As an initial test of whether firing rate was modulated during these latter movements, we scored all head and postural movements for a subset of 12 neurons and found that six neurons were excited during these movements (50%), while four were suppressed (33%), demonstrating that AId activity can be modulated during head and postural movements as well.

As indicated above, we observed both excited and suppressed responses within single neurons: 21 out of 68 neurons (31%) that responded during multiple movements exhibited excitation during some movements and suppression during others (Fig. 2*B*). The overall proportions of excited versus suppressed responses did not differ (56%, 138/247 excited; 44%, 109/247 suppressed; binomial test, *p* = 0.07), indicating a fairly even representation of excitation and suppression across scored movements. In addition, the proportions of excited versus suppressed responses during each movement type did not differ (binomial test, *p* > 0.05 in all cases; Fig. 2*C*).

### Temporal response patterns of AId neurons at movement onsets and offsets

**Figure 3.**
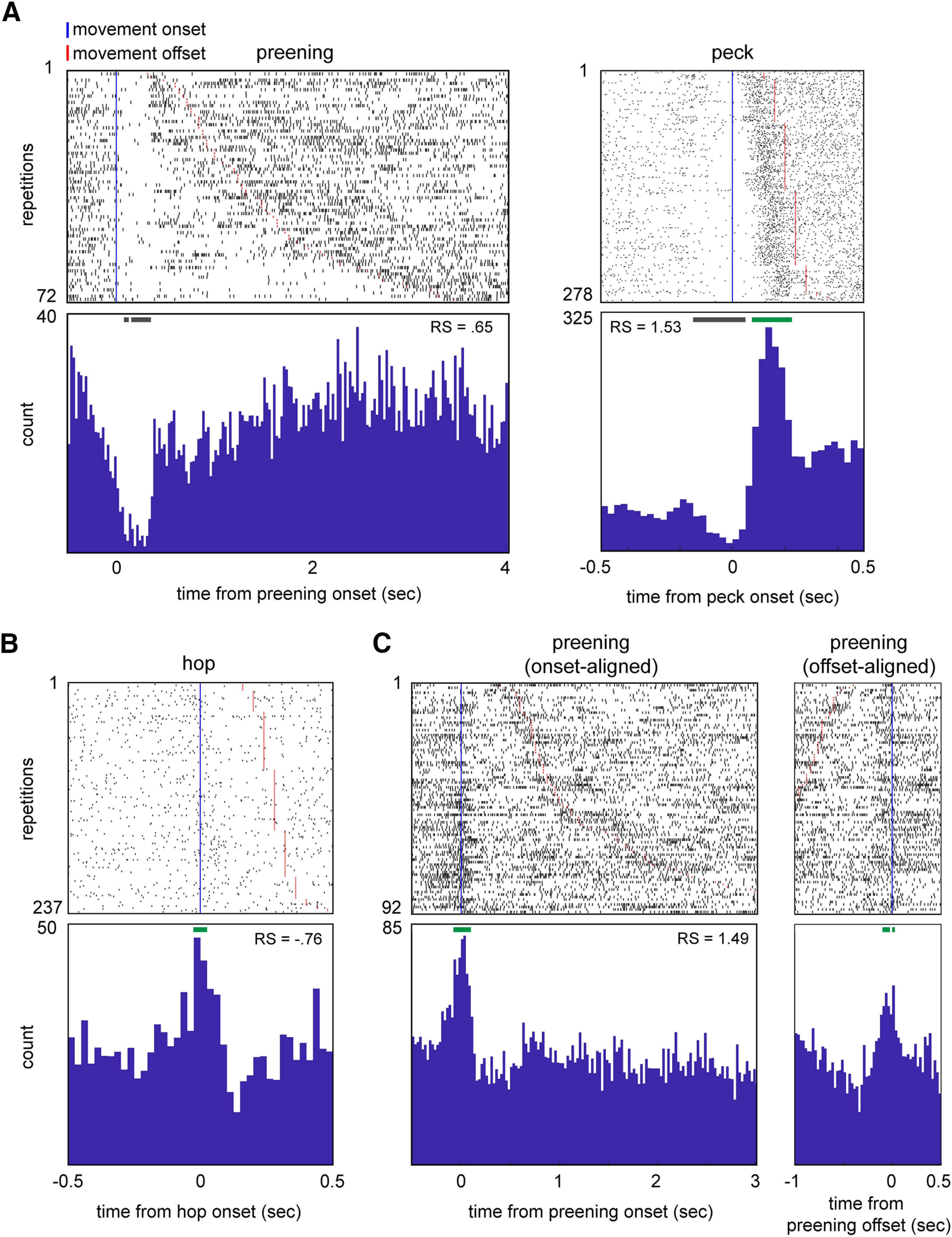
AId neurons show a variety of temporal response patterns during different scored movements. ***A***, Rasters and histograms illustrating the response of a single AId neuron during preening (left) and pecks (right). ***B***, Raster and histogram illustrating the response of a single AId neuron during hops. ***C***, Rasters and histograms illustrating the onset-aligned (left) and offset-aligned (right) preening response of an example AId neuron. Rows are sorted by movement duration. Blue vertical lines mark movement onsets; red lines mark movement offsets. Green and gray horizontal bars above histograms denote periods of excitation or suppression, respectively (see Materials and Methods). RS, average standardized response strength over the entire duration of each movement type.

Some AId neurons demonstrated consistent temporal changes in firing rate at movement onsets and/or offsets that could be masked by measures of average firing rate. For example, Figure 3*A* shows rasters and histograms for a single neuron during preening (Fig. 3*A*, left) and peck (Fig. 3*A*, right) responses. In both cases, mean firing rate during the movement was significantly excited relative to quiescence (preening RS = 0.65, peck RS = 1.53, *p* < 0.05 in both cases). However, the responses clearly contain periods of suppression that begin before movement onset.

To capture these firing rate modulations, we tested for significant excitation or suppression at movement onsets and offsets. For each response, we compared histograms of spiking activity centered on movement onsets or offsets to histograms of shuffled spike trains (25-ms bins) to identify bins with significant firing rate changes (see Materials and Methods). Onset or offset responses were defined as responses with two or more contiguous bins (50 ms) of significant excitation or suppression occurring within 100 ms of movement onset or offset. Figure 3 plots examples of onset-aligned (Fig. 3*A–C*, left) and offset-aligned (Fig. 3*C*, right) responses; green and gray horizontal bars above each histogram mark excited and suppressed bins, respectively.

We observed significant onset responses during pecking, hopping, and preening but not other movement types. The top of Table 1 lists onset responses for these three movements, classified by whether neurons showed a significant response based on average firing rate. Eleven out of 107 neurons (10.3%) exhibited significant excitation (7/11) or suppression (4/11) at peck onsets. Five out of 119 neurons demonstrated an onset response during hopping (4.2%; 4/5 excitation, 1/5 suppression), as did four out of 93 neurons during preening (4.3%; 3/4 excitation, 1/4 suppression). We also observed significant offset responses during pecking and preening: six out of 107 neurons exhibited pecking offset responses (5.6%; 4/6 excitation, 2/6 suppression), and three out of 93 neurons showed preening offset responses (3.2%; all excitation; Table 1, bottom).

**Table 1:** Proportions of onset and offset responses across all neurons for different movement types

	Peck-onset responses(±100 ms from peck onset)		Hop-onset responses(±100 ms from hop onset)		Preening-onset responses(±100 ms from preeningonset)	
Response basedon averagefiring rate	Excited(7)	Suppressed(4)	Excited(4)	Suppressed(1)	Excited(3)	Suppressed(1)
Excited	0.05 (5/107)	0.04 (4/107)	0.01 (1/119)	0	0.03 (3/93)	0.01 (1/93)
Not significant	0.02 (2/107)	0	0.02 (2/119)	0	0	0
Suppressed	0	0	0.01 (1/119)	0.01 (1/119)	0	0

	Peck-offset responses(±100 ms from peck offset)		Preening-offset responses(±100 ms from preeningoffset)			
Response basedon averagefiring rate	Excited(4)	Suppressed(2)	Excited(3)	Suppressed(0)		

Excited	0.04 (4/107)	0.01 (1/107)	0.01 (1/93)	0		
Not significant	0	0	0.01 (1/93)	0		
Suppressed	0	0.01 (1/107)	0.01 (1/93)	0		

Onset responses (top) and offset responses (bottom) are shown separately (total *n* = 29 responses), categorized based on whether average firing rate during the movement showed significant excitation (excited), suppression (suppressed), or no response (not significant).

A total of 13 of these 29 onset and offset responses did not match the average firing rate response (Table 1). For example, four responses (two pecking, two hopping) included consistent excitation at movement onset even though average firing rate during movement did not differ from quiescent baseline (Table 1, top, “not significant” row). Onset and offset responses could also differ in sign (excitation or suppression) from the average firing rate response: for example, while average firing rate during the hop response plotted in Figure 3*B* was suppressed relative to baseline (RS = –0.76), the raster and histogram reveal an excitatory peak starting just before hop onset, indicating a complex temporal response with brief excitation followed by suppression. These results suggest that single AId neurons can be modulated by multiple factors during movements, resulting in excitation at movement onsets or offsets and suppression during the movement itself, or vice versa.

### Context dependency of pecking behavior

Of the seven movements we scored, pecking behavior in particular tended to occur in different contexts: birds always pecked while eating, but also frequently pecked at other objects such as cage bars or perches. To investigate whether different contexts influenced responsivity, we compared RSs for pecks that occurred during eating versus non-eating.

**Figure 4.**
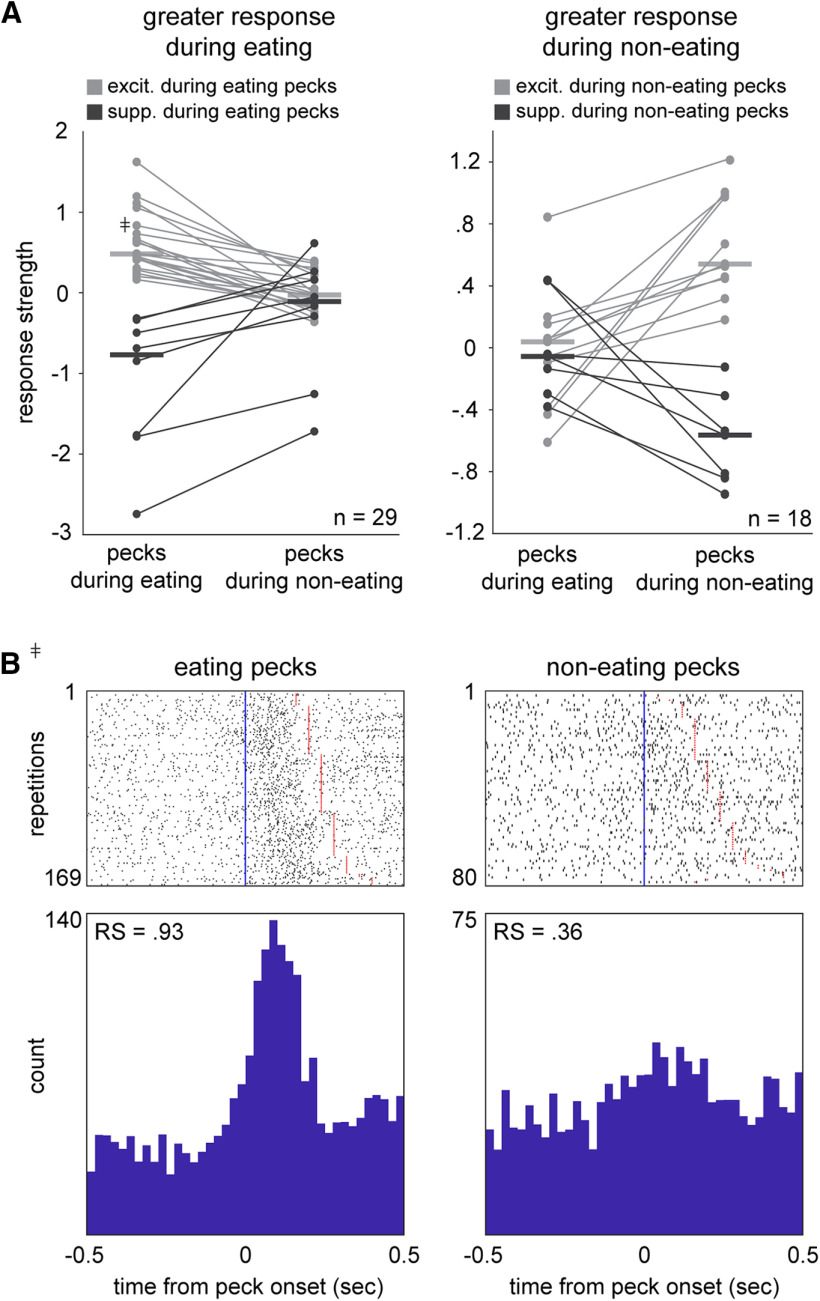
AId neurons exhibit context-sensitive peck responses. ***A***, Mean standardized RSs of neurons during pecks that occurred during eating versus non-eating periods, grouped by neurons that showed greater absolute RS during eating (left) and non-eating (right). Left, Gray and black lines represent neurons that showed positive or negative RS, respectively, during eating-pecks. Right, Gray and black lines represent neurons that showed positive or negative RS, respectively, during non-eating pecks. Horizontal bars represent medians. RSs during eating-related pecks were significantly different from non-eating pecks for all plotted neurons (Mann–Whitney tests). ***B***, Rasters and histograms of an example neuron’s response during pecks that occurred during eating (left) versus non-eating (right). Peck RS of this neuron is indicated by the cross-marked plot point in ***A***, left. Rows are sorted by peck duration. Blue vertical lines mark peck onsets; red lines mark peck offsets.

RS differed for eating- versus non-eating pecks in 47 out of 97 neurons (48%; Mann–Whitney test, *p* < 0.05). Figure 4*A* plots these context-sensitive cells according to whether they exhibited greater absolute RS during eating (29/47, 62%; left) or non-eating (18/47, 38%; right). Figure 4*A*, left panel, shows 29 neurons that exhibited greater absolute RS during eating-related pecks. Most of these neurons showed higher firing rates during eating-related pecks compared with non-eating pecks (21/29, 72%; gray lines). The peck-aligned activity for one of these neurons (Fig. 4*B*) illustrates strong excitation during pecks that occurred when the bird was eating (left) versus a weak response during non-eating (right). The remaining neurons showed lower firing rates during eating-related pecks compared with non-eating pecks (8/29, 28%; black lines). In contrast, Figure 4*A*, right panel, plots 18 neurons that showed greater absolute RS during non-eating pecks. Eleven of these neurons showed higher firing rates during non-eating pecks compared with eating pecks (61%, gray lines); the remaining cells showed lower firing rates during non-eating pecks (7/18, 39%, black lines).

These results indicate that pecking activity in many AId neurons was dependent on the context in which the movement occurred and suggest that neurons can signal behavioral contexts with either relative excitation or suppression. While peck duration did not differ between eating versus non-eating (mean peck duration = 0.24 ± 0.002 vs 0.22 ± 0.003 s, respectively), one possibility is that this context-dependent activity reflects differences in eating versus non-eating pecking movements. Alternatively, these differential responses may reflect that these neurons do not encode the physical movements of pecking behavior per se; for example, this subpopulation may be involved in processing orofacial or external sensory information that is present specifically in one context versus another.

### Singing-responsive neurons in AId

One of AId's primary sources of afferents is from LMAN-shell, which contains neurons that are active during singing behavior and have been implicated in guiding accurate imitation of the tutor song during vocal learning (Fig. 1*A*; Achiro and Bottjer, 2013; Achiro et al., 2017). Moreover, lesions of AId in juvenile birds impair their ability to achieve an accurate imitation of the adult tutor song without disrupting vocal motor output (Bottjer and Altenau, 2010; see Mandelblat-Cerf et al., 2014; Materials and Methods). Given this evidence of a role for AId in vocal learning, we hypothesized that the activity of AId neurons would be modulated as juvenile birds engaged in singing behavior.

**Figure 5.**
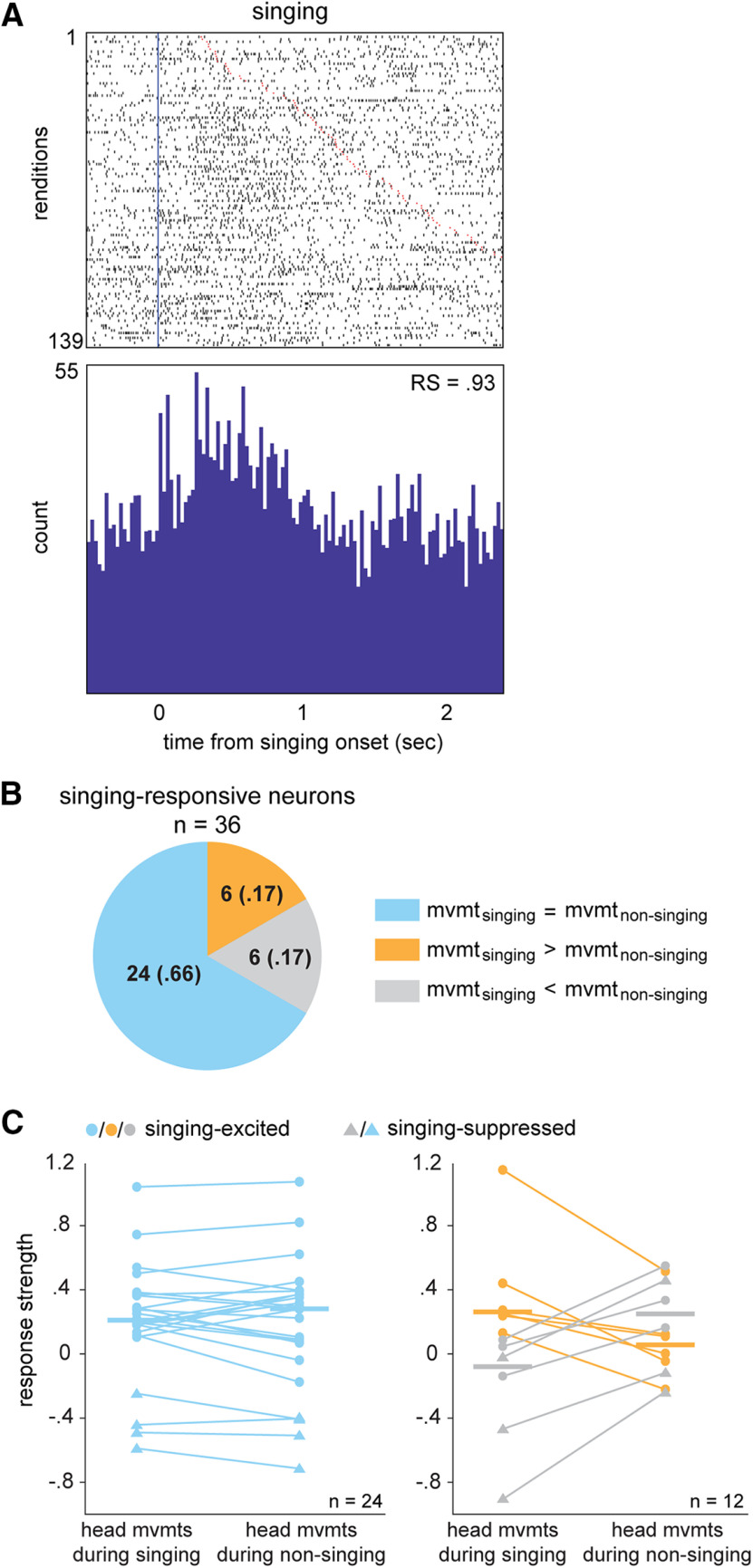
A substantial population of AId neurons are responsive during singing. ***A***, Raster and histogram illustrating activity of an example singing-excited AId neuron during singing episodes. Rows are sorted by duration of each singing episode. Blue vertical line marks onset of each singing episode; red lines mark ends of singing episodes. ***B***, Proportions of 36 singing-responsive neurons for which RS during head movements within singing periods was greater than (orange), less than (gray), or not different from (blue) head movements within non-singing periods. ***C***, Mean standardized RSs during head movements that occurred within singing versus non-singing periods. Left, Neurons that showed comparable RS during head movements that occurred within singing versus non-singing periods. Right, Neurons that showed higher RS during head movements that occurred within singing (orange) or non-singing (gray) periods. Lines connect data points from single neurons. Horizontal bars represent medians. Circles versus triangles represent neurons that showed an increase or decrease, respectively, in average firing rate across singing episodes relative to quiescence (see Tables 2-4).

Firing rates were significantly modulated during singing relative to quiescence in the majority of neurons (66/94, 70%), including 44 excited responses and 22 suppressed responses (mean RS = 0.76 ± 0.09 and –0.96 ± 0.19, respectively). Figure 5*A* illustrates the singing-aligned response of a neuron that was excited during song renditions. Altered firing rates during vocal production in songbirds have typically been interpreted as “singing specific.” However, birds often make head and postural body movements during singing, as well as beak-gape and gular-fluttering movements that are specific to song production. This complexity raises the question of which movements are an intrinsic part of singing behavior versus independent movements that are performed simultaneously during song production. Given the range of movement responsivity across AId neurons (Fig. 2), activity modulation in singing-responsive neurons may reflect singing-specific actions as well as movements that are performed during both singing and non-singing periods. As an initial test of this question, we compared neural activity during head movements that occurred within singing periods versus adjacent non-singing periods in a subset of 36 singing-responsive neurons. We then compared activity during singing periods with versus without head movements to assess whether head movements contributed to the singing response in these neurons (see Materials and Methods).

RS during head movements that occurred within singing versus non-singing periods did not differ for most singing-responsive neurons (24/36, 67%; Mann–Whitney test, *p* > 0.05 for each neuron; Fig. 5*B*,*C*, left). In eight of these 24 neurons, RS during singing periods that contained head movements was significantly greater than during singing that lacked head movements, indicating that activity during head movements contributed to the singing response (7/8 excited, 1/8 suppressed; Table 2, left). For the remaining 16 neurons, RS during singing that lacked head movements either did not differ from (14/24) or was greater than (2/24) RS during singing with head movements (Table 2, middle and right). In addition, most of these 24 neurons still showed significant changes in firing rates during singing that lacked head movements (21/24, 88%). Thus, firing rate changes in most of these singing-responsive neurons was not attributable to activity during head movements.

**Table 2:** Mean standardized RS during singing periods with versus without head movements (*n* = 24 neurons that showed comparable firing rates during head movements that occurred within singing and non-singing periods)

	Singing w/ headmovements > singing w/out head movements(*n* = 8/24)		Singing w/ headmovements = singing w/out head movements (*n* = 14/24)		Singing w/ headmovements < singing w/out head movements(*n* = 2/24)	
	Excited(7*)	Suppressed(1)	Excited(12)	Suppressed(2)	Excited(1)	Suppressed(1)
Singing w/ movements	0.47 ± 0.04	–1.75	1.08 ± 0.22	–0.67 ± 0.10	0.24	–0.74
Singing w/out movements	0.29 ± 0.03	–0.69	0.62 ± 0.10	–0.41 ± 0.17	0.49	–0.87

Neurons are categorized by whether RS during singing that included head movements was greater than (left), equal to (middle), or lower than (right) RS during singing that lacked head movements. Responses are significantly different from quiescence unless otherwise noted (*). Blue lines in Fig. 5C, left, depict head movement responses.

* 3/7 neurons were significantly excited during singing periods that contained head movements but not during singing periods that lacked head movements.

Six neurons were singing excited and showed greater RS during head movements that occurred within singing compared with non-singing (Fig. 5*B*,*C*, right, orange). In three of these neurons, activity during singing periods with head movements was greater than during singing without head movements, indicating that singing-specific head movements contributed to excitation during song production (Table 3, left). For the other three neurons, responses during singing periods with versus without head movements were comparable, suggesting that discrete head movements made little contribution to activity modulation during singing (Table 3, middle). Moreover, in all but one of these six neurons, activity during singing periods that lacked head movements was still significantly greater than quiescence.

**Table 3:** Mean standardized RS during singing periods with versus without head movements (*n* = 6 neurons that showed greater RS during head movements that occurred within singing compared with non-singing periods)

	Singing w/ headmovements > singing w/out head movements(*n* = 3/6)		Singing w/ headmovements = singing w/out head movements(*n* = 3/6)		Singing w/ headmovements < singing w/out head movements(*n* = 0/6)	
	Excited(3*)	Suppressed(0)	Excited(3)	Suppressed(0)	Excited(0)	Suppressed(0)
Singing w/ movements	0.84 ± 0.44		0.49 ± 0.08			
Singing w/out movements	0.47 ± 0.20		0.32 ± 0.09			

Neurons are categorized by whether RS during singing that included head movements was greater than (left), equal to (middle), or lower than (right) RS during singing that lacked head movements. Responses are significantly different from quiescence unless otherwise noted (*). Orange lines in Fig. 5C, right, depict head movement responses.

* 1/3 neurons was significantly excited during singing periods that contained head movements but not during singing periods that lacked head movements.

Three neurons were singing suppressed and showed lower RS during singing-related head movements compared with non-singing (Fig. 5*C*, right, gray triangles). For two of these neurons, decreased firing rates during head movements contributed to greater suppression during singing (Table 4, left, suppressed). However, all three neurons were suppressed even during singing periods that lacked head movements. In fact, for one of these neurons, this suppression was significantly greater than during singing that contained head movements (Table 4, right, suppressed). Interestingly, three neurons showed lower RS during singing-related head movements but nevertheless showed significant excitation across singing periods (Fig. 5*C*, right, gray circles; Table 4, excited columns), highlighting the presence of multiple modulating factors during song behavior.

**Table 4:** Mean standardized RS during singing periods with versus without head movements (*n* = 6 neurons that showed lower RS during head movements that occurred within singing compared with non-singing periods)

	Singing w/ headmovements > singing w/out head movements(*n* = 3/6)		Singing w/ headmovements = singing w/out head movements(*n* =1/6)		Singing w/ headmovements < singing w/out head movements(*n* = 2/6)	
	Excited(1)	Suppressed(2)	Excited(1)	Suppressed(0)	Excited(1)	Suppressed(1)
Singing w/ movements	0.35	–0.91 ± 0.70	0.44		0.44	–2.75
Singing w/out movements	0.10	–0.36 ± 0.34	0.24		0.74	–3.88

Neurons are categorized by whether RS during singing that included head movements was greater than (left), equal to (middle), or lower than (right) RS during singing that lacked head movements. All responses are significantly different from quiescence. Gray lines in Fig. 5C, right, depict head movement responses.

In summary, the singing-modulated activity of most neurons persisted in the absence of head movements (32/36, 89%; Tables 2-4). These results indicate that activity of many AId neurons during song production may reflect singing-specific movements such as respiratory actions, beak movements, or gular fluttering or non-physical aspects of song production such as auditory-vocal feedback. Interestingly, 12 neurons (33%) showed differential RS during head movements within singing versus non-singing periods (Fig. 5*B*,*C*, right). One interpretation is that these neurons integrate information about head movements and singing behavior, such that changes in firing rate are enhanced specifically during head movements that are performed concurrently with song. Developing associations between head or postural movements and vocal behavior may be a crucial component of learning to produce female-directed song and perform courtship dance movements during singing (Morris, 1954; Balaban, 1997; Williams, 2001; Tomaszycki and Adkins-Regan, 2005). These results raise the possibility that neural activity reflecting “non-singing” movements during song production may be a ubiquitous feature of circuits involved with song learning and control.

### Additional sources of AId neuron modulation: behavioral states

**Figure 6.**
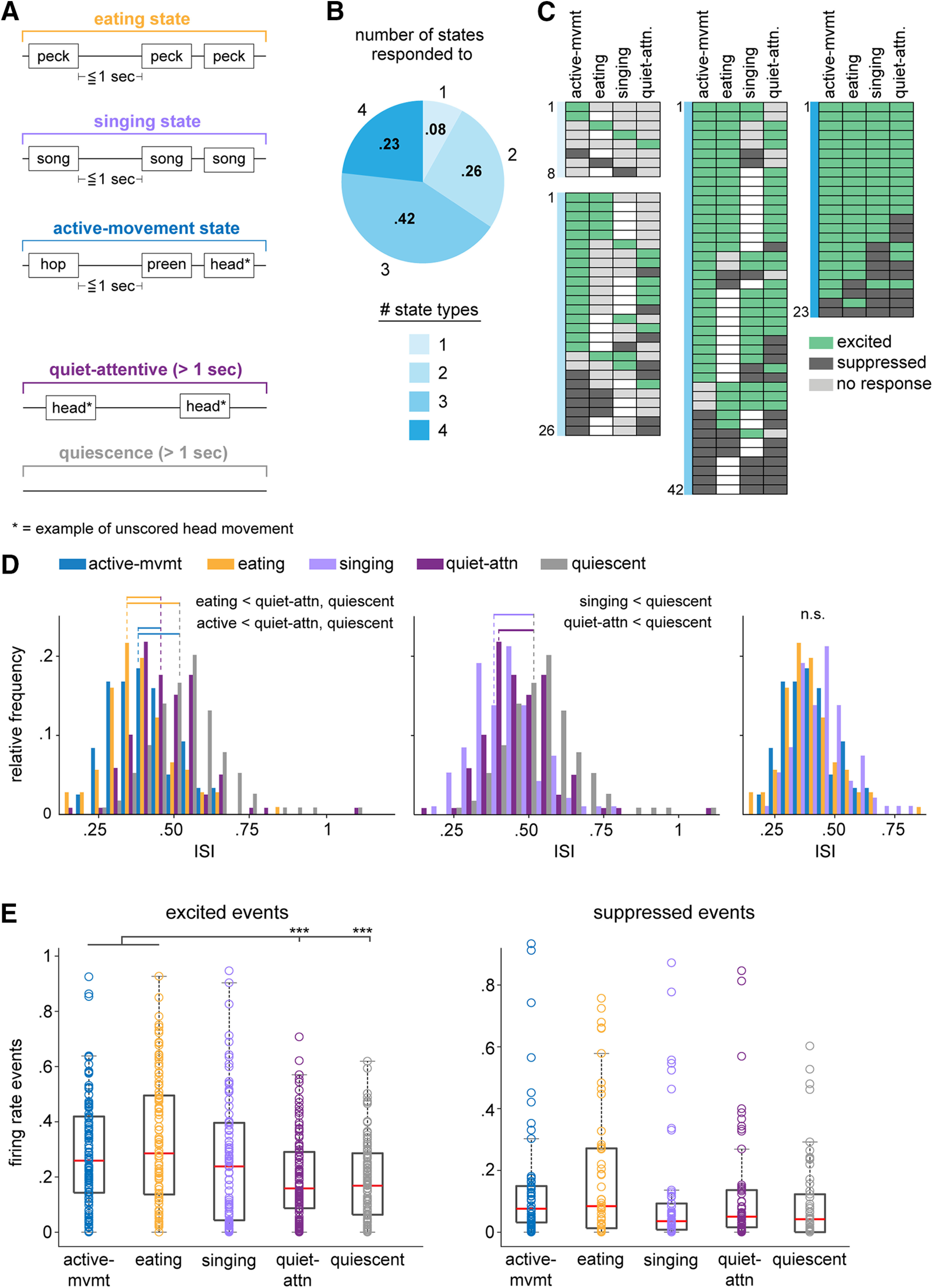
AId neurons are differentially modulated during different behavioral states. ***A***, Schematics of example eating, singing, active-movement, quiet-attentive, and quiescent states. Text boxes represent example scored and unscored (starred) movements that typically occurred within that state type, although other behaviors could also occur (see Materials and Methods). ***B***, Proportions of single AId neurons that were modulated during different numbers of state types. 8/99 state-responsive neurons were modulated during one state type, 26/99 during two, 42/99 during three, and 23/99 during four states. ***C***, Each row of each chart indicates the states during which each neuron was excited (green), suppressed (dark gray), or not responsive (light gray). Unfilled boxes indicate that no data during that state was recorded for that neuron. Charts are grouped according to colors in ***B***, based on the number of states during which activity of neurons was modulated. ***D***, Histograms comparing distributions of ISIs during active-movement, eating, quiet-attentive, and quiescent states (left); singing, quiet-attentive, and quiescent states (middle); active-movement, eating, and singing states (right). Horizontal lines indicate distributions that had significantly different means; dotted lines indicate means of the respective distributions. *p *<* *0.001 for all significant differences. ***E***, Number of excited (left) and suppressed (right) events that occurred during each state type, normalized by the total duration of each state type in a given recording session. Box-and-whisker plots indicate medians and first and third quartiles; whiskers indicate data points not considered outliers; circles represent data points from individual neurons; ***p <* *0.001.

As indicated above, our goal was to assess the activity of AId neurons throughout entire sessions of active behaviors. As part of this approach, we devised a novel way of characterizing each recording session by classifying contiguous time periods across each session into one of five different state periods based on the bird’s behavior: eating, singing, active-movement, quiet-attentive, or quiescence (Fig. 6*A*; see Materials and Methods). Eating states were defined as periods when the bird was engaged in eating behavior, including pecking at, hulling, and ingesting seeds; although eating state periods were dominated by eating-related behaviors, other movements such as head movements or hops could also occur. Similarly, singing states included periods when birds were engaged in song production, as well as brief pauses in-between song bouts during which birds occasionally hopped or pecked. During active-movement states, birds could produce any of the seven movements we scored as well as head and/or postural body movements that were not scored. The remaining two states characterized non-movement periods: during quiet-attentive states, the bird was alert and could make small head movements but was otherwise not moving; birds made no movements during quiescent states (quiescent states included periods from which baseline intervals were sampled in the scored-movement analyses above; see Materials and Methods).

For most neurons, firing rates during eating, singing, active-movement, and/or quiet-attentive state periods differed from quiescence (99/109, 91%; Kolmogorov–Smirnov test, *p* < 0.05). Few neurons showed changes in firing rate during only one state type; most neurons exhibited firing rate modulations during two or more states (Fig. 6*B*). Figure 6*C* illustrates responsivity of single neurons during each non-quiescent state type, categorized by the number of states during which each neuron’s activity was modulated. AId neurons could show increased or decreased firing rates during non-quiescent states, and many neurons were excited during one state type and suppressed during another (29/109, 27%). However, whereas single neurons were equally likely to be suppressed as excited during different discrete scored movements (Fig. 2*B*), modulation across entire state periods tended to be excitatory: within each state type, the proportion of neurons that were excited was significantly greater than the proportion that were suppressed (binomial test, active-movement and eating states *p* < 0.0001, singing and quiet-attentive states *p* < 0.05), and the overall proportion of excited state responses was greater than suppressed state responses (binomial test, *p* < 0.0001).

In accord with this pattern of results, ISIs during non-quiescent states were shorter than ISIs during quiescence (Fig. 6*D*, left, middle; pairwise linear contrasts, Benjamini–Hochberg corrected, *p* < 0.001 in all cases). In addition, ISIs during active-movement and eating states were shorter than quiet-attentive ISIs (*p* < 0.001 in both cases; Fig. 6*D*, left) and did not differ from singing-state ISIs (*p* > 0.05 in both cases; Fig. 6*D*, right). These results indicate greater increases in firing rate during periods of active behavior, particularly for active-movement and eating states.

To capture dynamic changes in activity across state periods, we identified excited and suppressed spiking events during each state type, defined as five or more contiguous 10-ms bins in which the firing rate exceeded the average quiescence firing rate by ≥3 SDs (for excited events) or fell below the average quiescence firing rate by ≤1.5 SDs (for suppressed events). On average, more excited events occurred within active-movement and eating states compared with quiet-attentive and quiescence states (pairwise linear contrasts, Benjamini–Hochberg corrected, *p* < 0.001 in all cases; Fig. 6*E*, left). The frequency of excited events during singing states was elevated relative to quiet-attentive and quiescence, but did not differ significantly from any of the other four states (pairwise linear contrasts, Benjamini–Hochberg corrected, *p* > 0.05 in all cases; Fig. 6*E*, left). The relatively modest incidence of excited events during singing compared with active-movement and eating states may indicate that firing rate modulation during singing states involves more uniform increases in tonic spike rate. The number of suppressed events did not differ between state types (pairwise linear contrasts, Benjamini–Hochberg corrected, *p* > 0.05 in all cases; Fig. 6*E*, right). Taken together, these results indicate an increase in firing rate during non-quiescent states, with a greater degree of excitatory modulation during active-movement, eating and singing states expressed as shorter ISIs as well as an increase in discrete high-firing periods during active-movement and eating states.

### How does activity during scored movements contribute to behavioral states?

**Figure 7.**
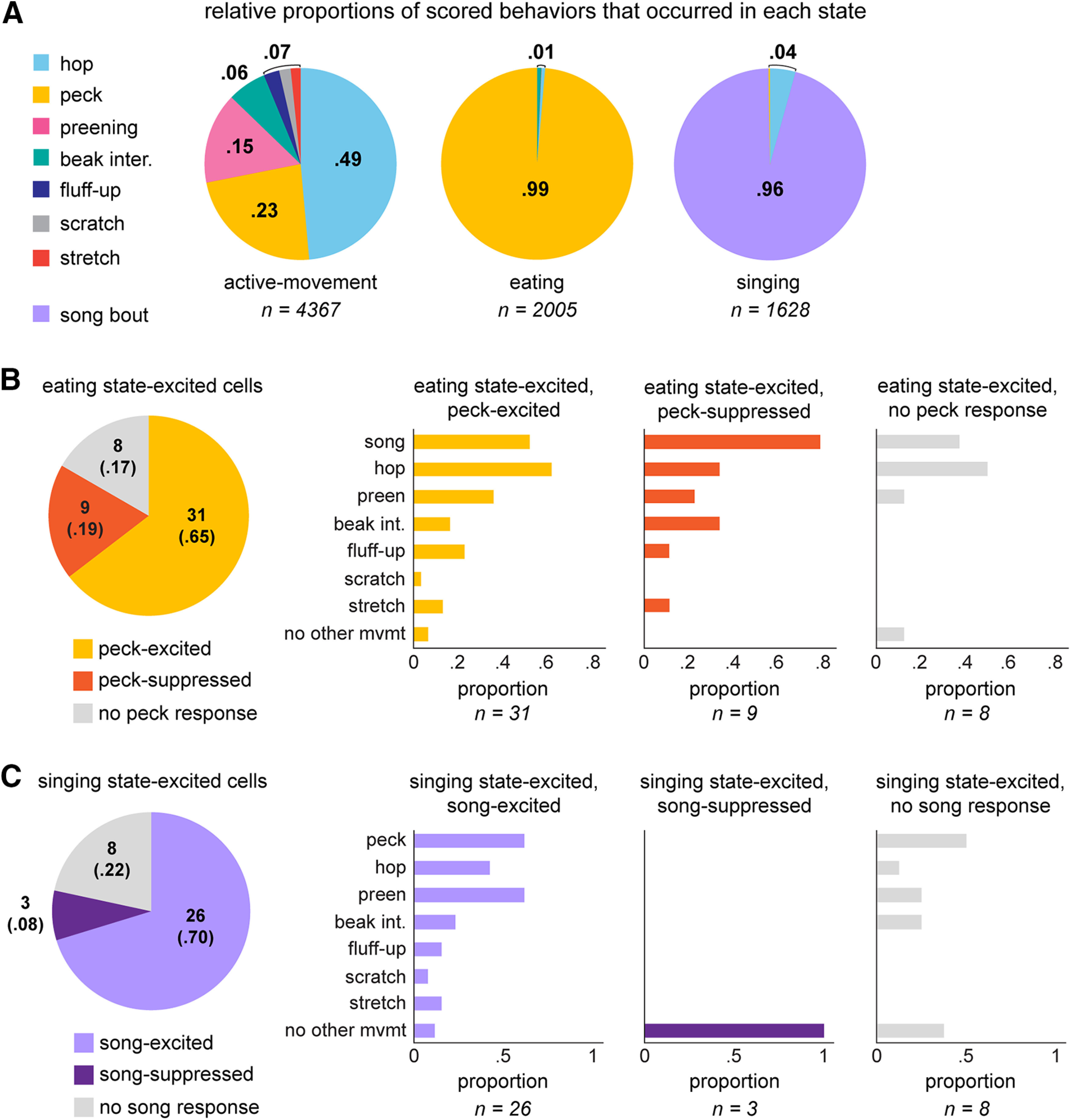
Movement-excited AId neurons are modulated by scored and unscored factors during different state periods. ***A***, Relative proportions of scored behaviors that occurred during each state type. Proportion totals include all occurrences of each of the seven scored movements as well as all song bouts. ***B***, left, Peck responsivity of 48 neurons that were significantly excited during eating state periods. Right, Proportions of eating-excited neurons that were responsive during scored behaviors, categorized by peck responsivity: eating-excited neurons that were peck-excited (left), peck-suppressed (middle), and unresponsive during pecks (right). “No other movement” represents neurons that did not respond during any other scored behavior. ***C***, left, Song-bout responsivity of 37 neurons that were significantly excited during singing state periods. Right, Proportions of singing state-excited neurons that were responsive during scored movements, categorized by song responsivity: singing state-excited neurons that were song-excited (left), song-suppressed (middle), and unresponsive during song bouts (right). “No other movement” represents neurons that did not respond during any other scored behavior. Italicized numbers indicate number of neurons within each song-responsivity grouping.

Because eating and singing state periods were characterized primarily by one type of scored movement (eating-related pecks and singing bouts, respectively; Fig. 7*A*), we wondered whether excitation during each of these states was restricted to neurons that were excited during the corresponding scored movements. If state responsivity of single neurons can be attributed to their movement responsivity, then neurons excited during eating states should also be excited during eating-related pecks, which accounted for 99% of scored movements during eating. Similarly, neurons excited during singing state periods should also be excited during song bouts. In contrast, if behavioral states include other sources of modulation, then we might expect to find a more diverse pattern of movement-related responsivity among eating-state-excited and singing-state-excited neurons; this latter outcome would be consistent with the heterogeneous pattern of movement responsivity described above.

Figure 7*B*, left, illustrates the peck responsivity of 48 neurons that were excited during eating states, grouped by their response during eating-related pecks. A majority of neurons that were excited during eating states were also excited during discrete peck movements (31/48, 65%). However, the remaining 35% of eating-state-excited neurons were suppressed or unresponsive during eating-related pecks, indicating that the heightened firing rate of these neurons during eating states did not relate to pecking behavior. Increased firing rates in these latter cells may be related to unscored factors during eating such as head movements or hulling behavior, or external sensory inputs. Likewise, most of the neurons that were excited during singing state periods were excited during song bouts (26/37, 70%), but the remaining 30% were suppressed or unresponsive during song bouts (Fig. 7*C*, left). Thus, excitation across singing states in these latter neurons presumably reflects activity during unscored factors that occur in-between song bouts.

Consistent with the fact that most neurons responded during multiple movements and states (Figs. 2, 6), neurons excited during eating and singing state periods were also responsive during a variety of scored movements that did not occur within these states. For example, many eating-state-excited neurons also responded during song bouts, preening, and fluff-ups, even though these behaviors never occurred within eating states (Fig. 7*A*,*B*, right). Similarly, most neurons excited during singing states were also responsive during scored movements that did not occur within singing states, such as preening, beak interactions, and fluff-ups (Fig. 7*A*,*C*, right).

These results highlight the complexity of information that AId neurons are processing. In many instances, single neurons were excited during a given behavioral state, but not during the scored movement that characterized that state. For example, some neurons were excited during eating states but suppressed during discrete eating-related pecks, indicating that the overall excitation seen across eating states was due in part to some other (non-pecking) influence. In addition, many neurons that showed excitation during a behavioral state were also responsive during multiple scored movements that were unrelated to that state. For example, an eating-state-excited neuron could also respond during movements that occurred outside of eating states, such as preening or fluff-ups. Thus, single neurons could demonstrate increased firing rates during state periods that were unrelated to scored movements, while also exhibiting modulation during specific movements that occur outside of that state type.

**Figure 8.**
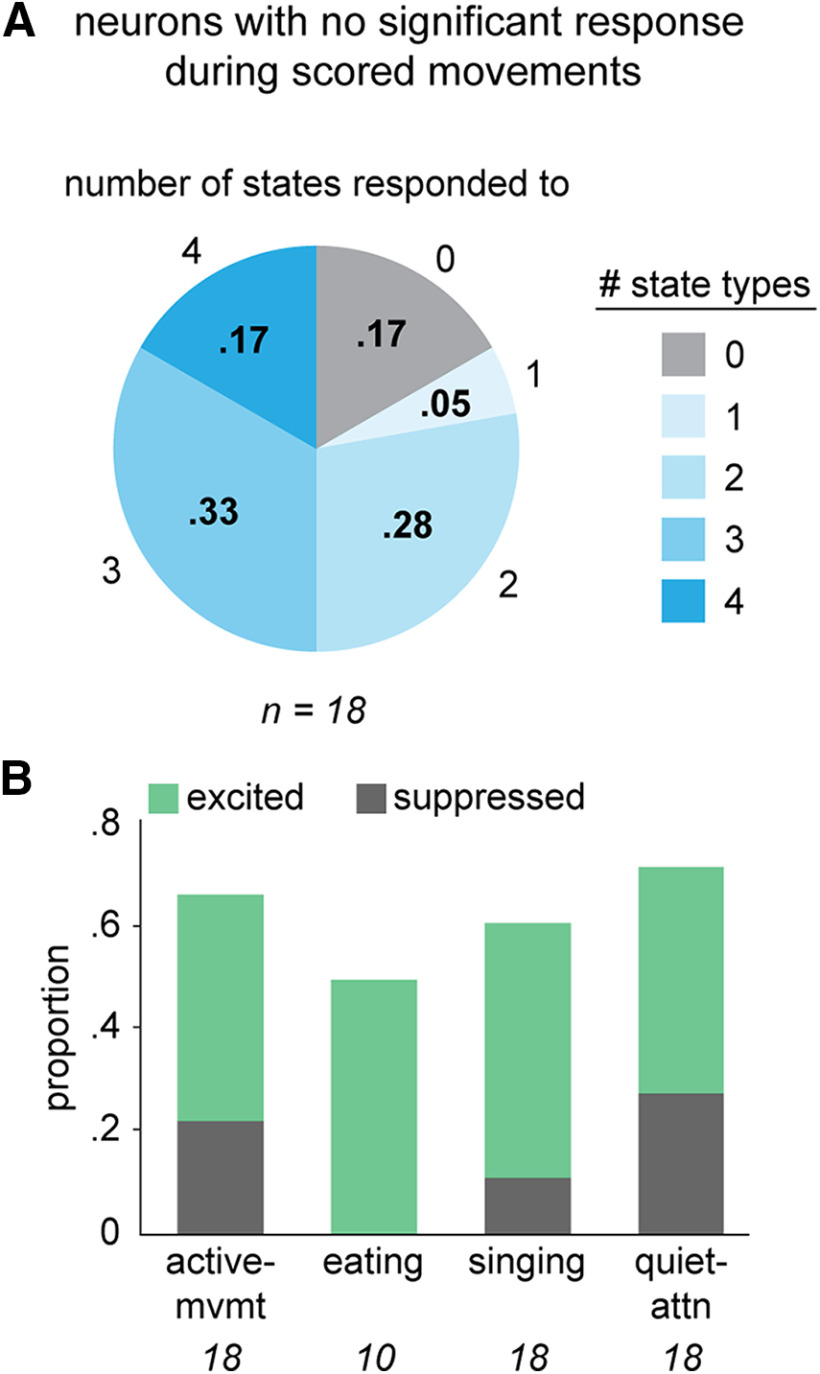
AId neurons that lack responsivity during scored movements are nonetheless modulated during different states. ***A***, Proportions of non-responsive AId neurons that were modulated during different numbers of state types. 3/18 neurons were not modulated during any states; 1/18 was modulated during one state type, 5/18 during two, 6/18 during three, and 3/18 during four state types. ***B***, Proportions of non-responsive neurons that were significantly excited (green) or suppressed (dark gray) during each state type. Italicized numbers indicate number of non-responsive neurons recorded during each state type.

In addition, while the majority of neurons we recorded were responsive during one or more scored movements (Fig. 2), 18 out of 119 cells (15%) were not significantly modulated during any scored movement. However, firing rates of most of these “movement-unresponsive” neurons were modulated during at least one state type compared with quiescence (15/18, 83%; Fig. 8). This result suggests that some of the neurons unresponsive during scored movements nonetheless exhibit firing rate changes related to unscored factors as the juvenile is actively behaving.

## Discussion

### Heterogeneous activity within AId reflects multidimensional tuning

We found that most AId neurons were selective for single movements or for different combinations of movements. Neurons responsive during different movements frequently demonstrated excitation during one movement and suppression during another. Moreover, individual responses could include transient excitation at movement onset and/or offset as well as suppression of average firing rate during the movement itself, or vice versa. The diversity of neural responses in AId is strikingly similar to the response profile of neurons in macaque motor cortex, where single neurons demonstrate heterogeneous, multiphasic temporal patterns of activity across reaching movements (Churchland and Shenoy, 2007). Such complex responses may result from multiple inputs relating to different movements or aspects of movements onto single AId neurons, as well as local transformation of afferent inputs. AId includes a local inhibitory network, evidenced by the fact that blocking GABA-A receptors in AId of anesthetized zebra finches elicits increased spontaneous firing rates, and parvalbumin expression is higher in AId compared with surrounding motor cortex (Mello et al., 2019; Yuan and Bottjer, 2019). Similarly, mammalian motor cortex contains a substantial population of inhibitory interneurons, which have been implicated in both regulating plasticity during motor skill learning and coordinating activity across motor cortex during behavior (Jacobs and Donoghue, 1991; Hess and Donoghue, 1994; Hess et al., 1996; Markram et al., 2004; Stagg et al., 2011; Donato et al., 2013; Chen et al., 2015; Kida et al., 2016; Adler et al., 2019). Blocking inhibition may unmask latent excitatory connections between spatially distant motor cortical neurons, providing a mechanism by which dynamic modulation of inhibition could flexibly reorganize connectivity and coordinate population activity across motor cortex (Jacobs and Donoghue, 1991; Spiro et al., 1999; Schneider et al., 2002; Capaday, 2004). AId receives topographic input from parallel circuits that process auditory, visual, and somatosensory information, so a similar mechanism to link different neuronal subpopulations within AId would be advantageous for facilitating sensorimotor integration across modalities (Zeier and Karten, 1971; Bottjer et al., 2000; Paterson and Bottjer, 2017).

The heterogeneous response profile of motor cortical neurons across taxa raises interesting questions about what factors contribute to the tuning of these neurons. Modulation of motor cortical activity has been associated with a variety of behavioral parameters in arm-reaching tasks, including movement direction, speed, trajectory, limb position, and joint angle (Evarts, 1968; Cheney and Fetz, 1980; Georgopoulos et al., 1982, 1988; Schwartz et al., 1988; Fu et al., 1993; Schwartz and Moran, 2000; Reina et al., 2001; Paninski et al., 2004; Churchland and Shenoy, 2007; Hatsopoulos et al., 2007). Increasing evidence indicates that multiple parameters can be reflected in the activity of single neurons, suggesting that integrated multimodal tuning may be a fundamental feature of motor cortical activity. For instance, recordings from macaques during unrestrained arm movements showed that parameters such as movement direction or end position of the limb could account for only a portion of spiking patterns in single motor cortical neurons, indicating that individual neurons may be tuned in a multidimensional space and that testing neural activity relative to any single parameter may account only partially for multidimensional tuning profiles (Ashe and Georgopoulos, 1994; Fu et al., 1995; Moran and Schwartz, 1999; Aflalo and Graziano, 2006, 2007). Likewise, we found that single AId neurons could be modulated both during diverse individual movements and during behavioral state periods that did not include those movements, indicating that single neurons were modulated by multiple factors.

Given convergent input from a diverse array of processing streams, the tuning profiles of neurons in both avian and mammalian motor cortex are not limited to motor responsivity. For instance, neurons in RA, which lies adjacent to AId in songbird motor cortex, drive vocal motor output in singing birds and demonstrate robust responses to playback of song stimuli in anesthetized or sleeping birds (Nottebohm et al., 1976; Doupe and Konishi, 1991; Vicario and Yohay, 1993; Wild, 1993; Yu and Margoliash, 1996; Dave et al., 1998; Dave and Margoliash, 2000; Leonardo and Fee, 2005; Kojima and Doupe, 2007; Sober et al., 2008; Yuan and Bottjer, 2019). Recordings from macaque motor cortex have likewise demonstrated sensitivity to non-motor stimuli: for instance, in visually-guided target-reaching paradigms, some motor cortical neurons exhibit selective activity related to the visual target, regardless of the limb trajectory used to reach that target (Tanji and Evarts, 1976; Evarts and Fromm, 1977; Murata et al., 1997; Shen and Alexander, 1997). The activity of AId neurons may be similarly modulated by integration of various factors to produce heterogeneous responses with complex temporal patterning during diverse movements. In support of this idea, we found neurons whose activity was modulated across behavioral state periods but not during any scored movements (Fig. 8). Although this activity could be related to head movements, which we did not score comprehensively, another possibility is that activity in these neurons relates to non-motor factors such as visual processing, attention, or arousal (Knudsen et al., 1995; Rauske et al., 2003; Cardin and Schmidt, 2004; Winkowski and Knudsen, 2007; Fernández et al., 2020).

### Multimodal integration provides behavioral context for voluntary movements

We found that most AId neurons demonstrated altered response strength during movements relative to quiescence; under the conditions of our recordings, firing rate modulations occurred most often during pecks, preening, and/or hops. It is difficult to know whether movement-related activity in AId neurons is pre-motor, modulatory, or reflective of movement feedback or external sensory inputs. AId neurons project to several targets, including the striatum, a dorsal thalamic zone, the lateral hypothalamus, a thalamic nucleus that relays to cerebellum [medial spiriform nucleus (SpM)], deep layers of the tectum, broad areas of the pontine and midbrain reticular formation, and VTA (Fig. 1*A*; Bottjer et al., 2000). The medial pontine reticular formation contains premotor neurons that contribute to neck and locomotive movements in other avian species (Steeves et al., 1987; Valenzuela et al., 1990; Dubbeldam, 1998; Wild and Krützfeldt, 2012); peck, preening, and hop-related activity may in part reflect these projections to premotor centers. Previous studies have found evidence of increased expression of the immediate early gene *egr-1* in AId specifically during hopping behavior (Feenders et al., 2008), which could indicate motor-related activity.

While these studies may indicate a role for AId in motor execution, other lines of evidence suggest that movement-related activity in many AId neurons is unlikely to reflect direct motor drive of peck, preening, and/or hopping behavior per se. Importantly, lesions of AId in juvenile birds do not disrupt song output or induce noticeable motor deficits, suggesting that AId neurons are not driving voluntary pecking or hopping movements (Bottjer and Altenau, 2010; see Mandelblat-Cerf et al., 2014; Materials and Methods). Moreover, the results presented here demonstrate that single AId neurons do not respond consistently during one particular type of movement. For instance, we found a substantial population of peck-responsive neurons that modulated their firing rate during pecks when the bird was eating but not when the bird pecked at other objects around the cage (or vice versa), even though pecking movements in these different contexts would recruit many of the same muscle groups. Furthermore, behavioral and functional experiments across avian species have implicated intermediate arcopallium in highly integrative, complex behaviors that extend beyond pure motor control, including ingestive behaviors, working memory processing, fear conditioning, and vocal learning (Lowndes and Davies, 1994; Lowndes et al., 1994; Knudsen and Knudsen, 1996; Aoki et al., 2006; Campanella et al., 2009; Saint-Dizier et al., 2009; Bottjer and Altenau, 2010; Achiro et al., 2017). One such example is a region comprising caudal arcopallium and nidopallium, which partially overlaps with AId and exhibited increased 2-deoxyglucose uptake when adult male zebra finches participated in their first courtship experience following several weeks of isolation from females; the amount of glucose consumption in this region correlated positively with isolation time but not with amount of movement activity (Bischof and Herrmann, 1986).

Rather than generically driving motor behavior per se, an important function of motor cortical circuitry is incorporating sensory information to appropriately direct motor output based on an animal’s environment and/or goals; this sensorimotor integration is necessary for voluntary movements such as goal-directed motor behaviors (e.g., object-directed grasping) as well as adaptive movements based on environmental perturbations. Avian and mammalian motor cortices receive multimodal inputs and target brainstem regions, making them ideally situated to carry out sensorimotor integration during voluntary behaviors. In macaques, neurons in motor cortical areas demonstrate a selective response when grasping at a particular object and corresponding visual selectivity for the same object when the monkey fixates on the object without grasping; inactivation of the same motor cortical region resulted in grasping deficits because of disrupted preparatory hand shaping that was inappropriate for the target object, suggesting a specific impairment in visuomotor transformations for targeted grasping rather than a gross motor impairment of hand movements (Murata et al., 1997; Fogassi et al., 2001; Rizzolatti and Luppino, 2001). Some AId neurons may serve a similar function in integrating sensory information to provide appropriate context for motor output. For example, the “eating-peck” responses observed here could represent an integrated response when the visual stimulus of seed is present as the bird pecks; these neurons could link visual information about seed with somatosensory information to contribute specifically to food-directed pecking behavior. In contrast, neurons that showed excitation during pecks associated with non-eating behaviors may process diverse inputs in the context of object exploration. Notably, some AId neurons could be involved in sensory-motor integration and also play a role in motor execution; likewise, other AId neurons could provide appropriate context for voluntary behaviors without directly driving motor actions. For instance, rather than directly linking sensory information to premotor centers, some neurons could instead feed multimodal information back into ascending reticular or tectal pathways to contribute to goal-directed behaviors, or differentially ascribe value to environmental cues depending on the animal’s current state or needs (Burgess et al., 2018).

### AId is uniquely situated to mediate learning and performance of both vocal and non-vocal elements of song behavior

Although motor cortex is generally well situated to integrate multimodal information related to a variety of goal-directed movements, AId’s unique connections suggest it may occupy a specific role in vocal learning and behavior. LMAN-core neurons that drive vocal motor output in juvenile birds make robust collateral projections into AId at 20–35 dph that substantially decline by 45 dph (Miller-Sims and Bottjer, 2012; J. H. Chung and S. W. Bottjer, unpublished observations). While our dataset did not include ages young enough to test the functional role of this connection between LMAN-core and AId, information from this developmentally regulated projection may play a critical role during the earliest stages of vocal practice and influence patterns of connectivity within AId that contribute to sensorimotor processing during subsequent learning. In addition, AId projects to higher-order thalamic nuclei that are linked to vocal learning, the dorsolateral nucleus of the medial thalamus (DLM) and the dorsomedial nucleus of the posterior thalamus (DMP; Fig. 1*A*; Bottjer et al., 2000). DLM is required for normal song behavior and projects to LMAN-shell, whereas DMP projects to the medial magnocellular nucleus of anterior nidopallium (MMAN); both LMAN and MMAN are required for development of an accurate imitation of tutor song (Bottjer et al., 1984; Foster et al., 1997; Vates et al., 1997; Foster and Bottjer, 2001; Aronov et al., 2008; Goldberg and Fee, 2011; Chen et al., 2014). AId also projects to lateral hypothalamus, striatum, and the area of dopaminergic neurons in VTA that projects to a nucleus in avian basal ganglia necessary for song learning (Fig. 1*A*; Bottjer et al., 2000); these limbic-related projections further suggest that AId neurons are well situated to contribute to vocal learning and behavior.

One hypothesis to integrate these unique connections with the multimodal integrative function of motor cortex is that some AId neurons may be involved in mediating learning and performance of movements in the context of song behavior. Song production in zebra finches is a courtship behavior during which males vocalize while performing a dance-like sequence of hopping movements oriented toward a female (Morris, 1954; Williams, 2001; Cooper and Goller, 2004; Dalziell et al., 2013; Ota et al., 2015; Ullrich et al., 2016); the temporal patterning of dance movements during song production is significantly correlated between father-son pairs of zebra finches, suggesting that non-vocal behaviors that accompany singing may be learned as well (Williams, 2001). Establishing a social context for courtship behavior likely involves sensory cues. For instance, adult males can use visual cues to select between two female birds shown in a silent video feed, but the addition of auditory cues induces stronger courtship responses (Galoch and Bischof, 2006, 2007). AId neurons are ideally positioned to integrate environmental context cues when females are present to guide learning and performance of movements that accompany singing.

In this framework, the relatively high proportions of peck, preening, and hopping-related responses observed here may reflect the fact that beak movements and hopping are important components of courtship behavior. In quail-chick chimeras, chicks that received transplants of lower brainstem somites from quails retained chick-like call structures but adopted quail-like patterns of head movements specifically during vocalizations; similar head movements made outside of vocalization periods were not affected, indicating the presence of specialized circuitry that mediates movements in the context of vocal behavior (Balaban, 1997). Circuitry processing non-vocal elements of singing in zebra finches may be similarly specialized; our results suggest that investigating how activity patterns during singing correspond to movements of different peripheral targets would benefit hypotheses for mechanisms of song production: for instance, we found a subpopulation of AId neurons that exhibited differential response strength during head movements that occurred during singing versus non-singing periods. These responses may indicate integration between non-vocal and vocal elements of song behavior, such that neural activity is enhanced specifically during head movements that accompany singing. This hypothesis raises an interesting prediction: although AId lesions in juvenile birds do not induce any gross motor deficits, it is possible that hopping and/or head movements performed during song production would be disrupted. Such a result would be consistent with previous studies in which c-Fos expression in AId was increased after adult male zebra finches performed non-singing courtship behaviors directed toward a live female (Kimpo and Doupe, 1997). Involvement of AId in courtship-related movements would draw an interesting parallel to mammalian studies that have suggested that motor cortex is parceled into “action zones” that each process information for different ethologically relevant categories of movement (Graziano, 2006; Graziano and Aflalo, 2007). For instance, stimulation of one region of macaque motor cortex results in the animal closing its hand in a grip while bringing it to its mouth and opening its mouth, as if eating an object, while stimulation of another region results in the monkey raising its arm and turning its head sharply to one side as if in defense (Graziano et al., 2002). In this context, adjacent motor cortical regions RA and AId could serve as an action zone that mediates the vocal and non-vocal movements that comprise song behavior.

While brainstem projections may mediate sensorimotor processing during vocal motor performance, the thalamic and midbrain projections of AId that give rise to recurrent feedback loops through cortico-basal ganglia circuitry may integrate multimodal information to facilitate song learning. Although AId does not drive song output (Bottjer et al., 2000; Bottjer and Altenau, 2010), we found a substantial population of singing-responsive neurons. For a large proportion of these neurons, the changes in firing rate during singing could not be attributed to any of the movements that we scored (including head movements). These firing rate modulations may instead be related to singing-specific movements such as beak movements or gular fluttering, or sensory activity such as auditory or proprioceptive feedback (Goller et al., 2004; Ohms et al., 2010; Bottjer and To, 2012; Riede et al., 2013). Alternatively, singing-related activity could reflect active evaluations of the juvenile’s vocal behavior during sensorimotor practice; iterative evaluations between self-generated output and the goal tutor song are essential for guiding accurate refinement of the juvenile’s song, and evidence of neural activity processing these comparisons has been reported in LMAN-shell, which projects directly to AId (Achiro et al., 2017). Importantly, successful song learning also requires multiple factors beyond simply matching vocal output to an auditory goal. For instance, vocal learning in juvenile zebra finches that are tutored with only passive playback of the tutor song is severely impaired, whereas pairing auditory tutoring with a visual model of an adult zebra finch enhances learning (Derégnaucourt et al., 2013; Ljubičić et al., 2016). Moreover, visual cues provided during singing, such as wing strokes or fluff-ups from adult females, provide feedback that can influence juvenile vocal learning (West and King, 1988; Morrison and Nottebohm, 1993; King et al., 2005; Carouso-Peck and Goldstein, 2019). Multimodal inputs from dNCL and singing-related inputs from LMAN may converge in AId, integrating important non-vocal and vocal elements of courtship song behavior that must be learned during a sensitive period of development.

## Synthesis

Reviewing Editor: Mark Laubach, American University Decisions are customarily a result of the Reviewing Editor and the peer reviewers coming together and discussing their recommendations until a consensus is reached. When revisions are invited, a fact-based synthesis statement explaining their decision and outlining what is needed to prepare a revision will be listed below. The following reviewer(s) agreed to reveal their identity: Jesse Goldberg, Todd Roberts.

Synthesis of Reviews

Two reviewers and I have reviewed your paper submitted to eNeuro. The consensus was to request major revisions. Details are provided in the following synthesis of our discussions.

Reviewer 1 described your study as follows:

"The authors studied motor cortical (AId) representations during natural behavior in juvenile zebra finches which are actively learning a complex motor sequence, the courtship song. They used extracellular recording of individual neurons in an area of the brain previously thought to be important for song learning, but not required for generating movements. They scored movement type by recording video simultaneously with neural data. A main result is that AId responses are highly heterogeneous with respect to movement types: individual neurons could be activated by some type of movement and suppressed by another, and show no rate change for yet other movements. This is consistent with findings from mammalian motor cortex. The novelty from this study is that the recording sessions encompass various naturally occurring behavior, rather than structured tasks. This study has the first awake-behaving recordings from AId, and thus an important characterization of its activities. The paper is strong and I have only minor issues.”

Essential revisions from this reviewer are:

1. The strength of the paper is its thorough analysis of neural activity and characterization of responses in different movement contexts. However how the movements are scored is not clear from the presentation. While the authors scored seven types of movements, they also report thousands of head and postural body movements that were not scored. What proportion of time are birds engaged in scored versus unscored movements? Is there significant overlap between these two (for example, hopping while moving head?). The best path forward for revision is to simply provide videos (even from an un-implanted bird) where different movement types are labeled.

EDITOR’S COMMENT: Both reviewers agree that provided video documentation of movement types is crucial.

2. Fig 1A, bottom: redundant with top plot.

EDITOR’S COMMENT: Please revise this figure to reduce redundancy across panels.

3. From line 385 ...pecking activity in many neurons was dependent on the context in which the movement occurred, suggesting that changes in response strength of these neurons is not specific to the physical movements of pecking behavior per se. This argument presumes the physical movements do not differ systematically between eating and other pecking. No evidence was provided for this assumption.

EDITOR’S COMMENT: Please provide evidence for movements being the same or different across conditions. Video recordings could be analyzed with software such as DeepLabCut to quantify the movements.

Reviewer 2 described your study as follows:

"In this manuscript the authors provide a somewhat detailed description of AId neuronal activity in juvenile male zebra finches during various behaviors, including eating, pecking, singing, hoping etc. They catalog widely heterogenous responses from this brain region. Most neurons are responsive while birds are performing multiple different behaviors. Many are responsive during eating, hoping and or singing, a smaller subset display context dependent changes in behavioral responses. I commend the authors on trying to examine the varied response properties of these neurons during equally varied natural behaviors. This research has the potential of providing a novel and valuable contribution to the understanding of activity in the arcopallium during behavior.

Unfortunately, it is not clear, at least as written, how this description advances our understanding of how motor skills like birdsong are learned or the role of AId in this process. The storyline of this manuscript is confusing and there seems to be a disconnect between what the results may indicate and how they both are introduced and discussed. Overall, I am left wondering what to take-away from this research.”

EDITOR’S COMMENT: In discussions among the reviewers and me, we came to the view that the novelty of the study is the report recordings of from AId. The study was characterized as descriptive and a novel advance. Please revise carefully based on the reviewer’s comments below.

Essential revisions from this reviewer are:

1. “The authors argue that some context dependent responses and modulations during different behavioral states indicate that AId is involved in integration of motor and sensory information rather than motor execution. This evidence and argument is not convincing, as many other stronger interpretations could be made. The problem here is that there are not independent compelling lines of evidence used to support these arguments.”

EDITOR’S COMMENT: Please revise the Discussion to address this comment.

2. “The Bottjer 2010 study showing lesions of AId disrupt song learning without disrupting singing ability is used to frame this study. One would think that the authors would seek to show how AId neurons are responsive to vocal errors or some other aspect of behavior that could be interpreted in the context of indirect aspect of motor performance or evaluation, as suggested by the Bottjer model. The context specific responses do not address this issue in any meaningful way.”

EDITOR’S COMMENT: Please revise the Discussion to address this comment.

3. “A peculiarity that considerably weakens the manuscript is that the authors almost completely ignore the paper from the Fee lab in eLife that claims to have lesioned this same area of the brain. This study claims that moderate lesions of Ad do not disrupt song learning while larger lesions of Ad result in severe disruptions in voluntary movements. It seems that the data presented here would be more easily interpreted from the perspective of AId being important for controlling these behaviors, rather than outright claiming that this brain region does not have any direct control of any of the behaviors under study. The circuit models from the Fee and Bottjer labs that can be used to frame and interpret the current data need to be directly and objectively addressed in this manuscript.”

EDITOR’S COMMENT: Please comment on the Fee study from eLife in the revised manuscript to address this issue.

4. “...there is no data showing the localization of recording sites and how they correspond to terminal fields from MAN or other known anatomical aspects of this area or the previous lesion studies.”

EDITOR’S COMMENT: Please provide anatomical localization data that references your recordings to terminal fields from MAN and previous lesion work.

5. “The cataloging of the various responses to various behaviors in the results section is lengthy and in places confusing, redundant, and some results seem contradictory. The description of neurons that are not responsive during movements but responsive during certain states is also confusing.”

EDITOR’S COMMENT: Please streamline the text describing response properties in the revised manuscript.

6. “The figures are not very intuitive and lack keys to identify what the colors mean (see pie charts for example).”

EDITOR’S COMMENT: Please revise figures to better identify the meaning of the colormaps.

7. “To a non-expert it is not clear why these studies have been conducted in juvenile birds rather than adults. Also, why not record in females for that matter?"

EDITOR’S COMMENT: Please comment on the decision to use juveniles and males in the revised manuscript. Methods section seems appropriate.

8. “Lastly, AId is referred to as motor cortex. AId is in the DVR and it is my understanding that it is not at all clear that neurons in this area of the brain are homologous to cortical neurons in general or motor cortex specifically. Seems that referring to them as motor pallium or some other similar term would be more accurate instead of this emphasis (even in the title) that this is motor cortex.”

EDITOR’S COMMENT: Please revise the Introduction to clarify that what you mean by motor cortex could also be characterized as motor pallium.

## Author Response

We thank the reviewers for their positive comments and thoughtful critiques, which we have used to make improvements throughout the paper.

We agree with the reviewers that video documentation is necessary to clarify the different movements we scored and the contexts in which they occurred. We have included two behavioral videos and a third that combines neural and behavioral recordings; together they demonstrate the seven different movements (including pecks during both eating and non-eating periods) as well as singing behavior, with the behaviors labeled as they occur. Unscored head and postural movements are apparent throughout the videos as well; as indicated in the Methods, head movements occurred so frequently that it was not possible to score them all. These movements did not occur concurrently with any of the seven scored movements, but frequently occurred in-between movements during non-quiescent periods and thus could have contributed to firing rate modulations across entire state periods (as noted in the “Behavioral scoring” section of the Methods and lines 482, 485, 487, and 547 of the manuscript). Head movements did occur during singing, which enabled us to analyze a subset of head movements during singing versus non-singing periods.

We have modified Figure 1 to include a schematic detailing the anatomical locations of each of our recording sites, as requested by Reviewer 2. In addition, we modified the figures to improve clarity: Reviewer 1 noted that Figure 1A included redundant pie charts - it is apparent that this reviewer was actually referring to Figure 2A; we agree that two pie charts are redundant and have therefore removed the top plot from both Figure 2A and 6B. We also added color keys (Figures 2, 4, 6) as suggested by Reviewer 2. In addition to including videos and improving the figures, we have also substantially edited the text to clarify and streamline the writing throughout.

We agree with Reviewer 1 that we cannot know whether pecking movements were identical between eating and non-eating behaviors. The idea that context-selective responses are unrelated to the motor act of pecking is only one interpretation of our results; we have revised the manuscript to clarify this point. As mentioned above, we have now included video examples of pecking movements in different contexts. As helpfully suggested by the Editor, we had tried using DeepLabCut to characterize different movements quantitatively. However, our video recordings were made with a single camera facing the front of the cage, and we found that even with DeepLabCut it was impossible to resolve consistently the 3D position of the bird’s head, beak, etc. during different movements with only a single plane of video. While quantitative characterization of movements would undoubtedly benefit future work such as investigation of AId firing in relation to specific movement dynamics, we believe that qualitative examination of these recordings serves as an important first step in associating AId activity with different naturally occurring behaviors.

Reviewer 2 expressed concern that the storyline of the manuscript was confusing and that the results do not advance our understanding of how motor skills like birdsong are learned. We agree that the rationale for the study was not well articulated and have revised the writing to clarify our approach. Although the Bottjer and Altenau 2010 lesion study clearly demonstrated that AId plays a role during sensorimotor learning, we respectfully disagree that the framing of the current study is solely dependent on that paper. In fact, we cite several papers from our lab showing that we (and others) had underestimated the anatomical complexity of the circuits that mediate vocal learning and behavior (e.g., Bottjer et al., 2000; Paterson and Bottjer, 2017). Correspondingly, we (and others) have had tunnel vision for targeting only song production, which limits our understanding of structure-function relationships in these pathways. We take an expanded view of vocal learning and performance - in addition to singing, other behaviors such as pecking, preening, and hopping frequently occur as part of courtship. Moreover, factors beyond auditory-vocal experience, such as social and visual cues, are important for accurate song learning. However, no previous chronic-recording studies that measured activity in any “song-system” brain region (including LMAN, HVC, RA, and others) have analyzed any behaviors other than singing; thus, we have no understanding of the range of behaviors that single neurons may encode. We believe the strength of this paper lies in the novel approach of measuring neural activity continuously throughout recording sessions to analyze activity during a diverse set of movements that are part of natural behavioral repertoires, including movements for courtship and learning. Our results document the multi-dimensional responsivity of AId neurons, showing that AId activity can be modulated not only during singing behavior but also during non-vocal behaviors; we consider these results to represent a goldmine of information that will aid experimental investigations of this region’s role in vocal learning, as well as provide a model for future studies of the diverse neural circuits that mediate acquisition of vocal motor skills in songbirds. We also think that studies in mammals could also benefit from a strategy of analyzing an expanded sample of diverse natural behaviors - not just artificially trained behaviors or a restricted single behavior.

Reviewer 2 expressed concern with our argument that AId is “involved in integration of motor and sensory information rather than motor execution.” We offer the idea that AId is involved in integration of motor and sensory information as one interpretation of our results; this idea is based in part on previous work from our lab showing that AId receives multi-modal sensory information (via dNCL, Fig. 1; Bottjer et al., 2000; Paterson and Bottjer, 2017). Even if AId neurons play some role in motor execution, the presence of these inputs strongly suggests that sensory-related information is integrated into that motor processing. In the Discussion, we reference the possibility that AId activity may relate to movement - for instance, the Feenders et al. 2008 study demonstrating ZENK activation in AId during hopping behavior, and the fact that one of AId’s projection targets includes brainstem regions that may overlap with premotor neurons for locomotor behavior (although it is possible that these projections target ascending, not descending, brainstem pathways). We also cite strong evidence indicating that AId neurons are not directly involved in motor execution: for instance, lesions of AId in juvenile birds do not disrupt song behavior and have no obvious effect on motor behaviors, and AId does not make any direct projections to known descending motor circuitry. Based on what is known about mammalian circuits, it is difficult if not impossible to identify cortical regions that are purely motor or purely sensory; consider the basic fact that the largest number of inputs to primary motor cortex come from primary somatosensory cortex - simple reach and grasp movements would be impossible without sensorimotor integration. Other song-system regions, including HVC, LMAN, and Area X (basal ganglia) show singing-related activity as well as time-aligned playback-evoked activity, showing that complex sensorimotor integration is a common feature of song-system circuits (McCasland and Konishi, 1981; Margoliash, 1983; McCasland, 1987; Doupe and Konishi, 1991; Vicario and Yohay, 1993; Yu and Margoliash, 1996; Doupe, 1997; Hessler and Doupe, 1999; Dave and Margoliash, 2000; Hahnloser et al., 2002; Leonardo and Fee, 2005; Prather et al., 2008; Achiro and Bottjer, 2013; Achiro et al., 2017; Yuan and Bottjer, 2019). We believe investigations of AId (and motor cortical function generally) would benefit from integrated consideration of both motor and non-motor factors, rather than an either-or framing.

Reviewer 2 suggests one of several interesting experiments that the Bottjer and Altenau 2010 lesion study leads to - namely, to investigate whether AId neurons demonstrate sensitivity to “vocal errors” or other markers of motor performance evaluation. Testing whether AId neurons respond to vocal errors is one of (many) future goals, and we mention that one possible interpretation of the singing-responsive neurons found here could be their involvement in vocal performance evaluation (lines 463-464, 781). However, while the Bottjer and Altenau study indicates that AId is necessary for accurate tutor song imitation, evaluation of motor performance is only one of several possible mechanisms by which AId could be involved in vocal learning. We addressed what we feel is a “prerequisite” issue in the current study, which is to determine if AId neurons are responsive not only during song behavior, but also during other diverse behaviors that are likely to be important components of song learning and courtship behavior. As we note above, previous investigations of the neural substrate of song behavior have analyzed neural activity only in relation to the act of singing, which may impose artificial restrictions on our knowledge of what “song-related” neurons are encoding. In addition to serving as the first demonstration that many AId neurons are active during singing, our results indicate that AId neurons are also modulated during a variety of other movements, suggesting that consideration of AId’s role in song learning may need to expand beyond auditory-vocal evaluations. We have revised the Introduction to emphasize our open approach, thereby helping to clarify why we analyze AId activity across a variety of movements rather than specifically testing song production only or sensitivity to vocal errors.

Reviewer 2 raised concerns about reconciling our interpretations of AId function with the results from the 2014 paper by Mandelblat-Cerf et al. in eLife. This reviewer suggests that our results could be interpreted based on the assumption that AId is important for controlling (unspecified) voluntary movements, “rather than outright claiming that this brain region does not have any direct control of any of the behaviors under study.” However, our lesions of AId (Bottjer and Altenau, 2010) did not reveal any motor disruption of song behavior or of any behaviors such as hopping, eating or preening - and certainly not “severe akinesia” after “complete” lesions as reported by Mandelblat-Cerf et al. This discrepancy alone between our results and theirs makes it clear that they were not lesioning the same region as what we define as AId. In our opinion, the study by Mandelblat-Cerf et al. is difficult to interpret for several reasons, many of which are detailed in the Note we posted for their article (https://hyp.is/LtYBegXREeirqUf3clFvKw/elifesciences.org/articles/02152), which we now reference in our manuscript. In regard to discrepancies in the impact of AId lesions on song learning, the explanation provided by Mandelblat-Cerf et al. is that our AId lesions had “an unintended impact on neurons in AIV.” However, it is difficult to evaluate their characterization of AIV in relation to AId. For one, it is very difficult to judge the location of their tracer injections into VTA/SNc (by which they defined AIV), and they never map out the distribution of corresponding retrograde label in Area X (or adjacent striatum) to confirm their injections. They report a “complex but consistent” pattern of label in an area that they identified as AIV, which clearly seems to correspond to a region called RA-cup that was previously documented by Darcy Kelly, Claudio Mello, and others (Kelley and Nottebohm, 1979; Vates et al., 1996; Mello et al., 1998; Gale et al., 2008). They claim that little or no projection from AId to VTA is revealed by their injections (see their Figure 1F), but do not mention our clear evidence for such a projection in Bottjer et al., 2000. In addition, they claim that they made lesions “to the portion of AId most likely affected by [their] AIV lesion procedure"; however, the AId lesion depicted in their Figure 5A (right) shows a very large lesion that did not include the medial-most aspect of AId (which is directly adjacent to RA and most likely to overlap with AIV) and instead encompassed much of the dorsolateral aspect of AId and a large amount of overlying cortex completely outside of the arcopallium. A lack of effect on vocal learning after this lesion would be consistent with the results described in Bottjer and Altenau showing that at least 80% of AId had to be lesioned in order to see a deficit in learning. Overall, it is unclear why Mandelblat-Cerf et al. were unable to replicate our carefully documented results in both the Bottjer et al. 2000 and the Bottjer and Altenau 2010 papers. We have cited all three papers and added a reference to our Note commenting on the Mandelblat-Cerf et al. paper, and suggest that interested readers can draw their own conclusions. In any case, the Mandelblat-Cerf et al. study indicates that motor disruptions were observed only after making more lateral lesions that would have extended beyond the lateral edge of LMAN-SHELL-recipient AId; the “moderate” lesions of AId described by Mandelblat-Cerf et al. did not induce motor disruptions, consistent with our reports that AId lesions do not disrupt motor behavior. As suggested by Reviewer 2, our revised manuscript includes anatomical localization of our recording sites, demonstrating that all of our recordings were sampled from within the intermediate arcopallial region in which lesions do not induce motor disruptions.

Reviewer 2 raised the important point that we gave no clear rationale for recording in AId of juvenile males, rather than adults and/or females. We chose to record in the early juvenile age range (43-58 dph) because this range corresponds to the early stages of sensorimotor integration (Immelmann, 1969; Böhner, 1983, 1990; Eales, 1985; Mann and Slater, 1995; Roper and Zann, 2006). We believe these recordings in juveniles lay important groundwork for future comparisons against recordings in adult birds to investigate, for instance, which components of AId activity relate to vocal learning versus performance. Females do not sing and we therefore chose to focus our recordings on males. We have revised the Methods to clarify these points.

We respectfully disagree with Reviewer 2’s comment regarding the reference to AId as motor cortex. We explicitly do not make any claims about homology at any point in the manuscript. Rather, we consider AId as analogous to motor cortex in connectivity and function; we use the term “cortex” in the generic sense as described by Reiner, Perkel, Bruce et al (2004, p. 395) as including that part of the telencephalon that is “pallial in nature and therefore homologous as a field to the brain region of mammals that includes the neocortex, claustrum, and pallial amygdala.” We have added this clarification to the Methods of the revised manuscript.

References

Achiro JM, Bottjer SW (2013) Neural representation of a target auditory memory in a cortico-basal ganglia pathway. J Neurosci 33:14475-14488.

Achiro JM, Shen J, Bottjer SW (2017) Neural activity in cortico-basal ganglia circuits of juvenile songbirds encodes performance during goal-directed learning. Elife 6:e26973.

Böhner J (1983) Song learning in the zebra finch (taeniopygia guttata): Selectivity in the choice of a tutor and accuracy of song copies. Anim Behav 31:231-237.

Böhner J (1990) Early acquisition of song in the zebra finch, Taeniopygia guttata. Anim Behav 39:369-374.

Bottjer SW, Altenau B (2010) Parallel pathways for vocal learning in basal ganglia of songbirds. Nat Neurosci 13:153-155.

Bottjer SW, Brady JD, Cribbs B (2000) Connections of a motor cortical region in zebra finches: relation to pathways for vocal learning. J Comp Neurol 260:244-260.

Dave AS, Margoliash D (2000) Song Replay During Sleep and Computational Rules for Sensorimotor Vocal Learning. Science 290:812-816.

Doupe AJ (1997) Song-and order-selective neurons in the songbird anterior forebrain and their emergence during vocal development. J Neurosci 17:1147-1167.

Doupe AJ, Konishi M (1991) Song-selective auditory circuits in the vocal control system of the zebra finch. Proc Natl Acad Sci U S A 88:11339-11343.

Eales LA (1985) Song learning in zebra finches: some effects of song model availability on what is learnt and when. Anim Behav 33:1293-1300.

Feenders G, Liedvogel M, Rivas M, Zapka M, Horita H, Hara E, Wada K, Mouritsen H, Jarvis ED (2008) Molecular mapping of movement-associated areas in the avian brain: A motor theory for vocal learning origin. PLoS One 3(3):e1768.

Gale SD, Person AL, Perkel ADJ (2008) A novel basal ganglia pathway forms a loop linking a vocal learning circuit with its dopaminergic input. J Comp Neurol 508:824-839.

Hahnloser RHR, Kozhevnikov A a, Fee MS (2002) An ultra-sparse code underlies the generation of neural sequences in a songbird. Nature 419:65-70.

Hessler NA, Doupe AJ (1999) Singing-Related Neural Activity in a Dorsal Forebrain-Basal Ganglia Circuit of Adult Zebra Finches. J Neurosci 19:10461-10481.

Immelmann K (1969) Song development in the zebra finch and other estrildid finches. In: Bird Vocalizations (Hinde RA, ed), pp 61-74. Cambridge University Press.

Kelley DB, Nottebohm F (1979) Projections of a telencephalic auditory nucleus-field L-in the canary. J Comp Neurol 183:455-469.

Leonardo A, Fee MS (2005) Ensemble coding of vocal control in birdsong. J Neurosci 25:652-661.

Mandelblat-Cerf Y, Las L, Denisenko N, Fee MS (2014) A role for descending auditory cortical projections in songbird vocal learning. Elife 3:e02152.

Mann NI, Slater PJB (1995) Song tutor choice by zebra finches in aviaries. Anim Behav 49:811-820.

Margoliash D (1983) Acoustic parameters underlying the responses of song-specific neurons in the white-crowned sparrow. J Neurosci 3:1039-1057.

McCasland JS (1987) Neuronal control of bird song production. J Neurosci 7:23-29.

McCasland JS, Konishi M (1981) Interaction between auditory and motor activities in an avian song control nucleus. Proc Natl Acad Sci U S A 78:7815-7819.

Mello C V, Vates GE, Okuhata S, Nottebohm F (1998) Descending auditory pathways in the adult male zebra finch (Taeniopygia guttata). J Comp Neurol 160:137-160.

Paterson AK, Bottjer SW (2017) Cortical inter-hemispheric circuits for multimodal vocal learning in songbirds. J Comp Neurol 525(15):3312-3340.

Prather JF, Peters S, Nowicki S, Mooney R (2008) Precise auditory-vocal mirroring in neurons for learned vocal communication. Nature 451:305-310.

Roper A, Zann R (2006) The Onset of Song Learning and Song Tutor Selection in Fledgling Zebra Finches. Ethology 112:458-470.

Vates GE, Broome BM, Mello C V, Nottebohm F (1996) Auditory pathways of caudal telencephalon and their relation to the song system of adult male zebra finches (Taenopygia guttata). J Comp Neurol 366(4):613-42.

Vicario DS, Yohay KH (1993) Song-selective auditory input to a forebrain vocal control nucleus in the zebra finch. J Neurobiol 24:488-505.

Yu AC, Margoliash D (1996) Temporal hierarchical control of singing in birds. Science 273:1871-1875.

Yuan RC, Bottjer SW (2019) Differential developmental changes in cortical representations of auditory-vocal stimuli in songbirds. J Neurophysiol 121:530-548.
